# Acupuncture combined with multiple therapies for angina pectoris: a systematic review and network meta-analysis

**DOI:** 10.3389/fcvm.2025.1463170

**Published:** 2025-01-30

**Authors:** Xiangyu Kong, You Gu, Zhao Qiu

**Affiliations:** ^1^ Department of Traditional Chinese Medicine, Shanyang Town Community Health Service Center, Shanghai, China; ^2^ Department of Traditional Chinese Medicine, Shanghai Pudong Hospital, Shanghai, China; ^3^ Department of Rehabilitation Medicine, Shanyang Town Community Health Service Center, Shanghai, China

**Keywords:** acupuncture, multiple therapies, angina pectoris, systematic review, network meta-analysis

## Abstract

**Objective:**

Acupuncture combined with multiple treatment modalities has been widely employed for treating angina pectoris. This paper compared the efficacy of acupuncture combined with multiple treatment modalities for angina pectoris by network meta-analysis (NMA).

**Methods:**

As of November 2023, this study searched eight electronic databases for randomized controlled trials (RCTs) of acupuncture combined with multiple modalities for the treatment of angina pectoris based on antianginal therapies. Primary efficacy indicators included the number of angina episodes and duration of episodes, and secondary indicators included clinical efficacy based on symptom improvement and electrocardiographic efficacy based on ST-segment and T-wave improvement. The Cochrane Risk of Bias tool 2.0 (RoB 2.0) was used for risk of bias assessment. A random-effects Bayesian NMA was performed using R (version 4.3.1) and Stata (version 16.0).

**Results:**

46 RCTs were enrolled, with 3976 patients with angina pectoris. In reducing the number of angina episodes, acupuncture [MD: −3.79; 95% CrI (−6.34, −1.31)] and acupuncture + TCM [MD: −3.06; 95% CrI (−5.49, −0.62)] were superior to antianginal therapies, with acupuncture having the best efficacy (SUCRA: 78.2%). In shortening the duration of angina episodes, electroacupuncture (EA) + traditional Chinese medicine (TCM) was the most effective (SUCRA: 95.1%), superior to antianginal therapies [MD: −5.04; 95% CrI (−9.18, −0.89)], adjunctive therapy [MD: 7; 95% CrI (1.58, 12.39)], rehabilitation therapy [MD: −5.38; 95% CrI (−10.75, −0.05)], and warm acupuncture + adjunctive therapy [MD: −6.71; 95% CrI (−13, −0.48)]. In terms of clinical efficacy, thumbtack needling had the best efficacy (SUCRA: 82.1%), superior to TCM [RR: 1.3; 95% CrI (1.02, 1.69)] and antianginal therapies [RR: 0.75; 95% CrI (0.6,0.91)]. In electrocardiographic efficacy, EA showed the best efficacy (SUCRA: 92.9%), superior to antianginal therapies [RR: 0.52; 95% CrI (0.35, 0.71)] and acupuncture [RR: 0.62; 95% CrI (0.39, 0.91)].

**Conclusion:**

Acupuncture performs best in reducing anginal episodes; EA + TCM is the most effective in shortening the duration of anginal episodes; thumbtack needling is the most effective in clinical efficacy; and EA shows optimal results in electrocardiographic efficacy. To further validate these findings, multicenter and large-sample RCTs are needed.

**Systematic Review Registration:**

PROSPERO [CRD42024505456].

## 1 Introduction

Cardiovascular disease (CVD) is the leading cause of death globally. In 2019, 27% of global deaths were caused by CVD. In 2020, CVD accounted for 48.00% and 45.86% of deaths in the rural and urban areas in China, and the projected number of people living with CVD in China was 330 million, of which 11.39 million had coronary heart disease. In 2021, CVD was responsible for approximately 19.91 million deaths worldwide, and nearly half of Americans (48.6%) suffered from CVD (1, 2). Angina pectoris, a typical symptom of CVD, especially coronary artery disease, limits mobility and professional or leisure activities and has a serious impact on patients’ quality of life. The presence of angina may indicate progression of coronary artery disease, increasing the risk of myocardial infarction, heart failure, and other cardiac events (3, 4). Current clinical guidelines provide recommendations for antianginal therapy. However, it is often difficult to control symptoms with a single agent, and multiple agents are needed to maximize efficacy and avoid adverse effects. In patients with specific comorbidities, antianginal medications are limited by drug suitability, side effects, and individual differences. Patients still experience persistent angina symptoms after adequate treatment with first- and second-line drugs (3, 5). On the other hand, percutaneous coronary intervention (PCI) can consistently improve clinical symptoms in patients with stable angina who have severe coronary stenosis without antianginal medication. However, the efficacy of PCI in improving exercise capacity and angina symptoms has been questioned in patients receiving the best combination of pharmacologic regimens (6, 7). Moreover, 20%–40% of patients may still experience angina in the short term despite optimized PCI therapy, which places a burden on their quality of life and finances (8). Therefore, the search for safe, cost-effective, and individualized treatment strategies is the focus of current research.

Acupuncture is an integral component of traditional Chinese medicine (TCM) with a history spanning thousands of years. By stimulating specific acupoints, it achieves therapeutic effects in treating diseases and alleviating pain. The efficacy of acupuncture has been recognized by the international medical community and has been confirmed in diverse diseases (9, 10). Acupuncture and its related therapies are widely applied clinically for angina pectoris in China as a nonpharmacologic treatment. Several meta-analyses to date have reported the efficacy of acupuncture in reducing angina attack frequency. Compared to angina medication alone, acupuncture yielded better results in improving patients’ anxiety and depression as well as better performance on the six-minute walk test, suggesting that it may improve patients’ physical fitness, mental health, and cardiac function (11–13). A randomized controlled clinical trial (RCT) by Zhao et al. revealed that electroacupuncture (EA) reduced the frequency and pain intensity of angina and improved quality of life (14). Multiple treatment modalities such as thumbtack needling, warm acupuncture, moxibustion, and acupuncture + TCM have been reported to have positive efficacy for angina (15–17). Among them, thumbtack needles are small needling tools that apply weak and prolonged stimulation to specific acupoints and are used to stimulate the skin surface without penetrating into the muscle or deeper tissue. These findings emphasize the potential and value of acupuncture combined with multiple therapeutic modalities as a nonpharmacological treatment for angina pectoris.

Clinicians frequently base their selection of an acupuncture approach for angina on their personal experiences, leading to significant variability in the treatment methods used by different physicians and resulting in diverse patient outcomes. A direct comparison of the effectiveness of acupuncture combined with multiple treatment modalities for angina is currently lacking. Therefore, we conducted a network meta-analysis (NMA) to evaluate and compare the efficacy of acupuncture combined with multiple treatment modalities for angina. Using the SUCRA score, we ranked the approaches based on number of episodes, duration of episodes, clinical efficacy, electrocardiographic (ECG) efficacy, TCM symptom score, and nitroglycerin use to assist health professionals and clinicians in making evidence-based decisions.

## 2 Method

### 2.1 Design and registration

This NMA followed the PRISMA statement (18) and was registered in the PROSPERO (CRD42024505456).

### 2.2 Search methods

PubMed, Embase, the Cochrane Central Register of Controlled Trials, Web of Science, CNKI, VIP, Wanfang Data, and SinoMed were independently searched by two researchers (K.X.Y. and G.Y.) up to 7 November 2023, without restrictions in study type, date, and publication status. MeSH terms and free words of keywords, including “Angina Pectoris”, “Angina, Stable”, “Angina, Unstable”, “Acupuncture”, and “acupuncture therapy” were utilized for article retrieval. The searching strategies are displayed in Supplementary Table S1. We further looked for references to related literature to prevent omissions.

### 2.3 Inclusion and exclusion criteria

The eligibility criteria were set based on PICOS principles, and the inclusion criteria covered (1) patients with angina pectoris of any age; (2) intervention: at least one of the following intervention modalities (any form, dose, or duration): acupuncture, EA, warm acupuncture, thumbtack needling, or a combination with these interventions. These acupuncture interventions were studied most frequently in patients with angina pectoris. (3) All patients received baseline antianginal therapies according to guideline recommendations, which included β-blockers, aspirin, statins, and angiotensin-converting enzyme inhibitors. Pharmacologic treatment was tailored to each patient. On this basis, additional adjunct (Adj) therapies were introduced in some control and intervention groups, respectively. (4) outcome: at least one of the following indexes: number of episodes, duration of angina episode, clinical symptom improvement rate, ECG improvement rate, TCM symptom score, and nitroglycerin use. The rates of clinical improvement and ECG improvement are defined in Supplementary Tables S2, S3. (5) The study design was an RCT. The language of these studies was limited to English or Chinese. Among them, only those Chinese studies in the “Core Journals of Peking University” or “Chinese Scientific and Technical Journal Database” were included. Articles were excluded for (1) unclear diagnostic and efficacy criteria; (2) reviews, cohort studies, case reports, opinions, descriptive studies, or abstracts; (3) incomplete or erroneous data that could not be combined; (4) publication before 2013.

### 2.4 Study selection

Study selection was performed independently by two investigators (K.X.Y. and G.Y.) according to our predefined criteria. First, all retrieved studies were imported into EndNote 20 to remove duplicates. Then, titles and abstracts were initially screened to eliminate ineligible RCTs. Finally, the full text of the initial screened matches was further screened to identify the final included literature. Disagreements were addressed through discussion with a third researcher (Q.Z.).

### 2.5 Data extraction

The following data and information were extracted: (1) basic information such as title, first author's name, year of publication, country, and study type; (2) basic characteristics of the study population, including age, gender, number of cases, and disease type; (3) specific content of the intervention, including specific measures and time schedules; and (4) baseline and post-treatment outcome metrics, such as number of anginal episodes, duration of anginal episodes, clinical efficacy, ECG efficacy, TCM symptom score, and nitroglycerin use. One investigator (K.X.Y.) extracted the data, and then the accuracy was confirmed by another investigator (G.Y.).

### 2.6 Quality assessment

The risk of bias was judged by two independent reviewers (K.X.Y. and G.Y.) using the Cochrane Risk-of-Bias Tool Version 2 (RoB 2.0) (19). Each trial was rated as “low risk”, “some concerns”, or “high risk” across the following domains: randomization; deviations from intended interventions; missing data; outcome measurement; and selective reporting of results. The trials were rated as an overall high risk of bias if one or more domains were rated as “high risk of bias” and as an overall low risk of bias if all domains were rated as “low risk of bias”.

### 2.7 Data synthesis

Statistical models based on the Bayesian framework were constructed using the JAGS software (gemtc 0.8–2 and rjags 4–10 package) in R 4.3.1 (Rstudio, Boston, MA, USA). Continuous data were depicted as mean difference (MD) with a 95% credible interval (CrI) to determine the effect size, while categorical data were presented as risk ratio (RR) with a 95% CrI. Random-effect models were utilized for all NMA due to the clinical heterogeneity in the included trials. The surface under the cumulative rank curve (SUCRA) was employed to assess the relative ranking of various modalities for each outcome. The ranking of interventions is positively correlated with higher SUCRA values (20). Furthermore, the comparison between consistency and inconsistency models was conducted through deviation information criterion (DIC). A discrepancy of <5 points in DIC indicated good consistency, leading to the utilization of consistency modeling (21). To examine publication bias, funnel plots were employed. Network plots and funnel plots for NMA were generated using Stata 16.0 (StataCorp, College Station, Texas, USA).

## 3 Result

### 3.1 Retrieval results

**Figure 1:**
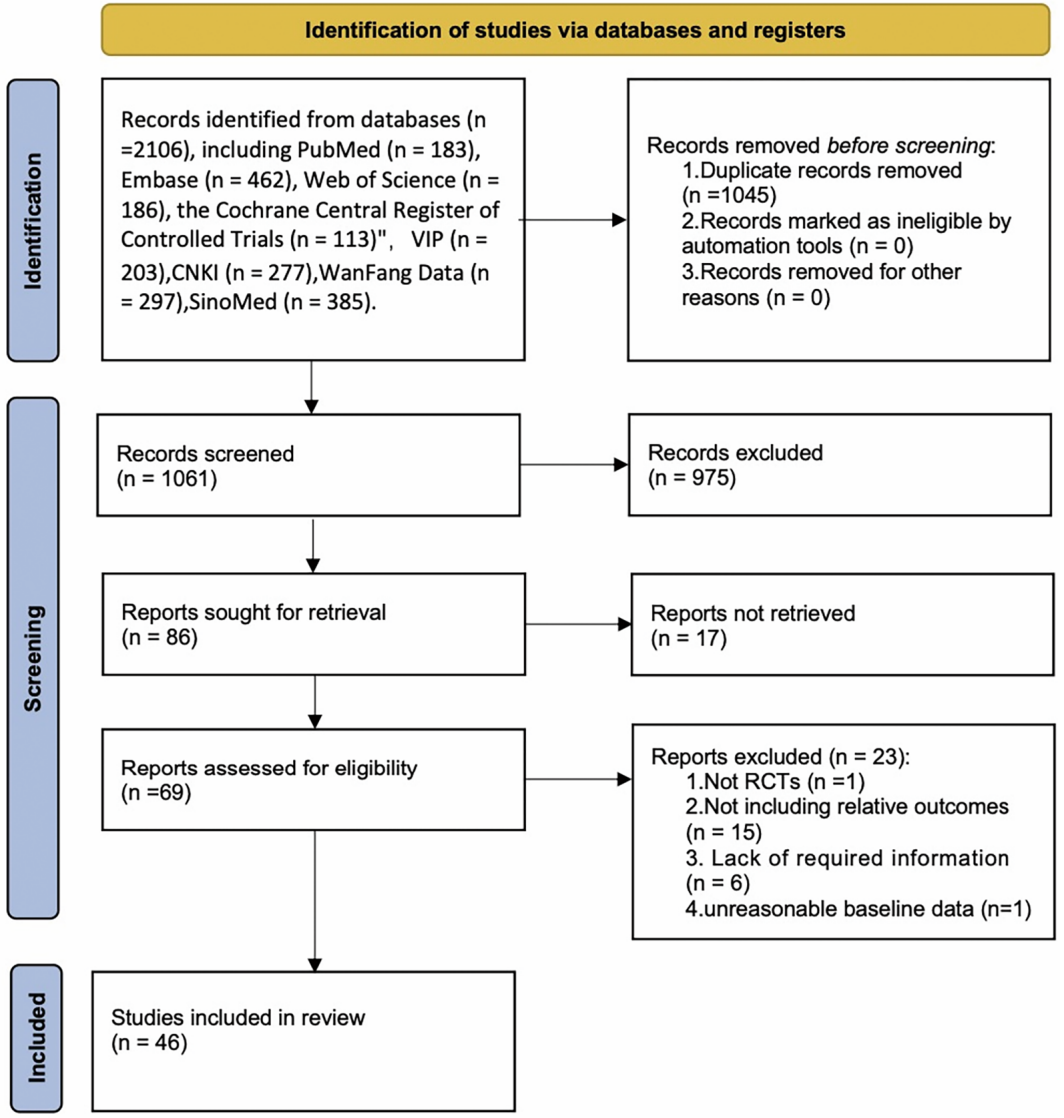
PRISMAT flowchart for the NMA.

The detailed procedure is displayed in Figure 1. Initially, 2,106 articles were retrieved. After removal of 1,045 duplicates, the title and abstract of each article were screened. Consequently, 975 articles were excluded. Following further full-text examination, 17 articles that did not have a full text and 23 articles that did not meet the criteria for RCTs and lack of data were eliminated. Ultimately, 46 articles were enrolled in the NMA.

### 3.2 Characteristics of enrolled studies

The details of each eligible article are displayed in Table 1 (Supplementary Table S4). 46 eligible studies published in 2013–2023 were all conducted in Asia (15–17, 22–64), enrolling 3,976 patients with angina pectoris, with study sample sizes ranging from 45 to 147 and a mean age of 44.7–73.16 years. Among them, 9 articles recruited patients with unstable angina (29, 34, 37, 38, 47, 53, 57, 62, 63), 6 recruited patients with coronary heart disease with angina (28, 33, 41–43, 51), 2 enrolled patients with type 2 diabetes mellitus with angina (25, 32), 1 recruited elderly patients with coronary heart disease, angina pectoris, and hyper homocysteine (46), 1 recruited patients with angina pectoris (56), 1 recruited patients with chronic stable angina pectoris (26), 1 recruited female patients with syndrome X (54), and others 27 trials recruited patients with stable angina pectoris. All patients received antianginal therapies, and 2 trials used EA (15, 51), 1 trial used EA + TCM (42), 2 trials used thumbtack needling (15, 48), 3 trials used warm acupuncture (29, 35, 54), 1 trial used acupuncture + acupressure (33), 1 trial used acupuncture + Adj therapy + moxibustion (16), 2 trials used acupuncture + moxibustion (17, 46), 1 trial used acupuncture + rehabilitation (24), 1 trial used acupuncture + TCM + moxibustion (17, 41), 1 trial used acupuncture + TCM + topical patch (49), 1 trial used acupuncture + TCM injection (36, 37), 1 trial used acupuncture + TCM injection and TCM oral administration (59), 15 trials used acupuncture (22, 25, 27, 32, 38, 40, 43, 53, 55, 57, 58, 60, 61, 63, 64), and 19 trials used acupuncture + TCM (22, 23, 26, 28, 30, 31, 34, 39, 43–45, 47, 50, 52, 55, 56, 60–62). Among the 46 trials, 6 trials involved 3 groups (15, 55, 58, 60, 61, 63), 2 trials involved 4 groups (14, 40), and the remaining 40 trials involved 2 groups. Seven efficacy indices were evaluated, namely clinical efficiency (*n* = 41) (15–17, 22–39, 41, 44–57, 59, 60) (62–64), ECG efficacy (*n* = 14) (15, 25, 44–46, 48–51, 57, 59, 60, 62, 63), number of angina attacks (*n* = 19) (16, 22–26, 28–31, 35, 38, 40, 42, 43), (46, 52, 53, 58), duration of anginal attacks (*n* = 16) (16, 22–26, 28–30, 35, 38, 42, 43, 46, 47, 52), TCM symptom score (*n* = 10) (25, 27, 28, 30, 31, 39, 41, 44, 49, 50), Nitroglycerin use (*n* = 8) (25, 30, 38, 40, 43, 47, 53, 58).

**Table 1:** Main characteristics of the studies included in the network meta-analysis.

Author (Year)	Participant types	Sample size (Male)		Age (Years)		Intervention		Outcomes
		Control	Treatment	Control	Treatment	Control	Treatment	
Chen et al. (2023) (15)	SAP	30 (18)	30 (18)/30 (14)	71.30 ± 7.50	68.63 ± 9.84/71.57 ± 7.89	Antianginal therapies	EA/Needle-embedding	a, b
Sun et al. (2023) (17)	SAP	40 (24)	39 (19)	59.27 ± 5.14	61.33 ± 6.07	Acupuncture + Moxibustion	Acupuncture + TCM + Moxibustion	a
Zhang et al. (2023) (16)	SAP	41 (23)	40 (21)	58 ± 7	59 ± 7	Adj therapy	Acupuncture + Adj therapy + Moxibustion	a, c, d
Li et al. (2022) (22)	SAP	25 (17)	25 (15)	47.23 ± 4.28	48.04 ± 4.21	Acupuncture	Acupuncture + TCM	a, c, d
Zheng et al. (2022) (23)	SAP	44 (27)	44 (29)	62.01 ± 7.71	61.93 ± 7.93	TCM	Acupuncture + TCM	a, c, d
Fu et al. (2022) (24)	SAP	32 (16)	33 (18)	58.51 ± 4.95	58.43 ± 4.87	Rehab	Acupuncture + Rehab	a, c, d
Jiang et al. (2021) (25)	T2DM complicated with CHD and AP	37 (19)	37 (17)	65.87 ± 5.22	65.15 ± 5.13	Antianginal therapies	Acupuncture	a, b, c, d, e, f
Du et al. (2021) (26)	CSAP	60 (33)	60 (37)	63.3 ± 6.6	63.5 ± 7.5	Antianginal therapies	Acupuncture + TCM	a, c, d
Sun et al. (2021) (27)	SAP	58 (33)	58 (34)	60.62 ± 3.47	60.89 ± 2.45	Antianginal therapies	Acupuncture	a, f
Liu et al. (2021) (28)	CHD with AP	52 (29)	52 (32)	65 ± 4	65 ± 5	TCM	Acupuncture + TCM	a, c, d, f
Zhang et al. (2021) (29)	UAP	40 (30)	40 (28)	61 ± 6	62 ± 5	Antianginal therapies	Warm Acupuncture	a, c, d
Liu et al. (2021) (30)	SAP	30 (20)	30 (18)	53 ± 5	54 ± 6	Antianginal therapies	Acupuncture + TCM	a, c, d, e, f
Ding et al. (2021) (31)	SAP	40 (24)	41 (24)	62.80 ± 6.03	62.59 ± 5.73	Antianginal therapies	Acupuncture + TCM	a, c, f
Wang et al. (2021) (32)	T2DM with SAP	63 (40)	64 (42)	49 ± 10	50 ± 10	Antianginal therapies	Acupuncture	a
Gao et al. (2020) (33)	CHD with AP	51 (31)	51 (30)	62.03 ± 6.38	62.10 ± 6.40	Antianginal therapies	Acupuncture + Acupressure	a
Wu et al. (2020) (34)	UAP	43 (27)	43 (29)	59.57 ± 8.36	59.64 ± 8.42	Antianginal therapies	Acupuncture + TCM	a
Ye et al. (2020) (35)	SAP	58 (37)	58 (35)	67 ± 7	67 ± 6	Antianginal therapies	Warm Acupuncture	a, c, d
Zhang et al. (2020) (36)	SAP	72 (38)	74 (41)	63.16 ± 8.63	61.71 ± 8.49	TCM Injection	Acupuncture + TCM Injection	a
Wang et al. (2019) (37)	UAP	62 (39)	62 (32)	51.28 ± 3.12	53.07 ± 2.35	Antianginal therapies	Acupuncture + TCM Injection	a
Pan (2019) (38)	UAP	49 (33)	49 (31)	63 ± 9	63 ± 10	Adj therapy	Acupuncture	a, c, d, e
Wang et al. (2019) (39)	SAP	42 (27)	42 (25)	61.84 ± 8.50	62.40 ± 8.11	Antianginal therapies	Acupuncture + TCM	a, f
Zhang et al. (2019) (40)	SAP	30	30/30/30	NR	NR	Antianginal therapies	Acupuncture	c, e,
Wu et al. (2019) (41)	CHD with AP	46 (28)	46 (27)	66.59 ± 6.25	66.55 ± 6.23	Antianginal therapies	Acupuncture + TCM + Moxibustion	a, f
Chen et al. (2019) (42)	CHD with AP	30 (13)	30 (14)	62 ± 5	62 ± 5	TCM	EA + TCM	c, d
Li et al. (2019) (43)	CHD with AP	45 (19)	51 (22)	71.69 ± 24.03	73.16 ± 23.17	Acupuncture	Acupuncture + TCM	c, d, e
Gong et al. (2018) (44)	SAP	40 (22)	40 (28)	51.25 ± 5.48	53.57 ± 6.06	Antianginal therapies	Acupuncture + TCM	a, b, f
Chen et al. (2018) (45)	SAP	48 (28)	52 (35)	66.21 ± 2.48	62.38 ± 2.23	Antianginal therapies	Acupuncture + TCM	a, b
Sun et al. (2018) (46)	Senior CHD with AP complicated by HCY	44 (24)	44 (26)	62.73 ± 5.74	61.42 ± 4.86	Antianginal therapies	Acupuncture + Moxibustion	a, b, c, d
Lu et al. (2018) (47)	UAP	43 (29)	46 (30)	51.2 ± 6.2	51.9 ± 6.5	Antianginal therapies	Acupuncture + TCM	a, d, e
Deng et al. (2018) (48)	SAP	38 (22)	38 (27)	56.49 ± 11.36	57.12 ± 9.78	Antianginal therapies	Needle-embedding	a, b
Deng et al. (2017) (49)	SAP	30 (15)	30 (16)	62.4 ± 2.3	63.2 ± 2.6	Antianginal therapies	Acupuncture + TCM + Topical Patch	a, b, f
Shi et al. (2017) (50)	SAP	35 (21)	35 (20)	60.17 ± 5.61	59.97 ± 7.06	Antianginal therapies	Acupuncture + TCM	a, b, f
Wang et al. (2017) (51)	CHD with AP	45 (30)	45 (28)	62.11 ± 5.42	61.51 ± 4.95	Antianginal therapies	EA	a, b
Wu et al. (2017) (52)	SAP	57	57	NR	NR	Antianginal therapies	Acupuncture + TCM	a, c, d
Yan et al. (2017) (53)	UAP	47 (26)	47 (27)	63.7 ± 4.0	63.4 ± 4.3	Adj therapy	Acupuncture + TCM	a, c, e
Wang et al. (2016) (54)	CSX	40	40	53 ± 5	53 ± 5	Antianginal therapies	Warm Acupuncture	a
Jia et al. (2016) (55)	SAP	30	30/30	NR	NR	Antianginal therapies	Acupuncture/Acupuncture + TCM	a
Fu et al. (2016) (56)	AP	35 (22)	35 (21)	56.7 ± 10.2	56.4 ± 10.5	Antianginal therapies	Acupuncture + TCM	a
Li et al. (2015) (57)	UAP	21 (5)	30 (9)	44.7	46.7	Antianginal therapies	Acupuncture	a, b
Wang et al. (2015) (58)	SAP	15 (5)	15 (7)/15 (6)	56	59/57	Healthy controls	Antianginal therapies/Acupuncture	c, e,
Jin et al. (2015) (59)	SAP	36 (14)	36 (17)	59.37 ± 9.26	60.23 ± 10.56	TCM Injection	Acupuncture + TCM Injection + TCM + TCM	a, b
Huang et al. (2014) (60)	SAP	20 (11)	20 (7)/20 (7)	59.95 ± 7.49	59.30 ± 6.82/60.55 ± 7.81	Acupuncture	TCM/Acupuncture + TCM	a
Xie et al. (2014) (61)	SAP	20 (11)	20 (7)/20 (7)	59.95 ± 7.49	59.30 ± 6.82/60.55 ± 7.81	Acupuncture	TCM/Acupuncture + TCM	b
Zhao et al. (2013) (62)	UAP	30 (14)	30 (15)	58.6 ± 5.4	61.4 ± 5.8	Antianginal therapies	Acupuncture + TCM	a, b
Jin et al. (2013) (63)	UAP	36 (14)	36 (16)/36 (15)	57.13 ± 12.14	54.15 ± 13.15/58.41 ± 13.2	Antianginal therapies	Topical Patch/Acupuncture	a, b
Qiu et al. (2013) (64)	SAP	40 (25)	40 (24)	62.5	63.2	Antianginal therapies	Acupuncture	a

All patients received baseline antianginal therapies. Only additional interventions beyond the baseline treatment are listed in the table. NR, not report; TCM, Traditional Chinese Medicine; AP, angina pectoris; CSX, cardiac syndrome X; SAP, stable angina pectoris; UAP, unstable angina pectoris; T2DM, type 2 diabetes mellitus; CSAP, chronic stable angina pectoris; CHD, coronary heart disease; HCY, homocysteine; Adj therapy, adjunctive therapy; EA, electroacupuncture; a, clinical efficacy rate; b, ECG efficacy rate; c, frequency of episodes; d, duration of episodes; e, nitroglycerin dosage; f, TCM syndrome score.

### 3.3 Quality assessment

**Figure 2:**
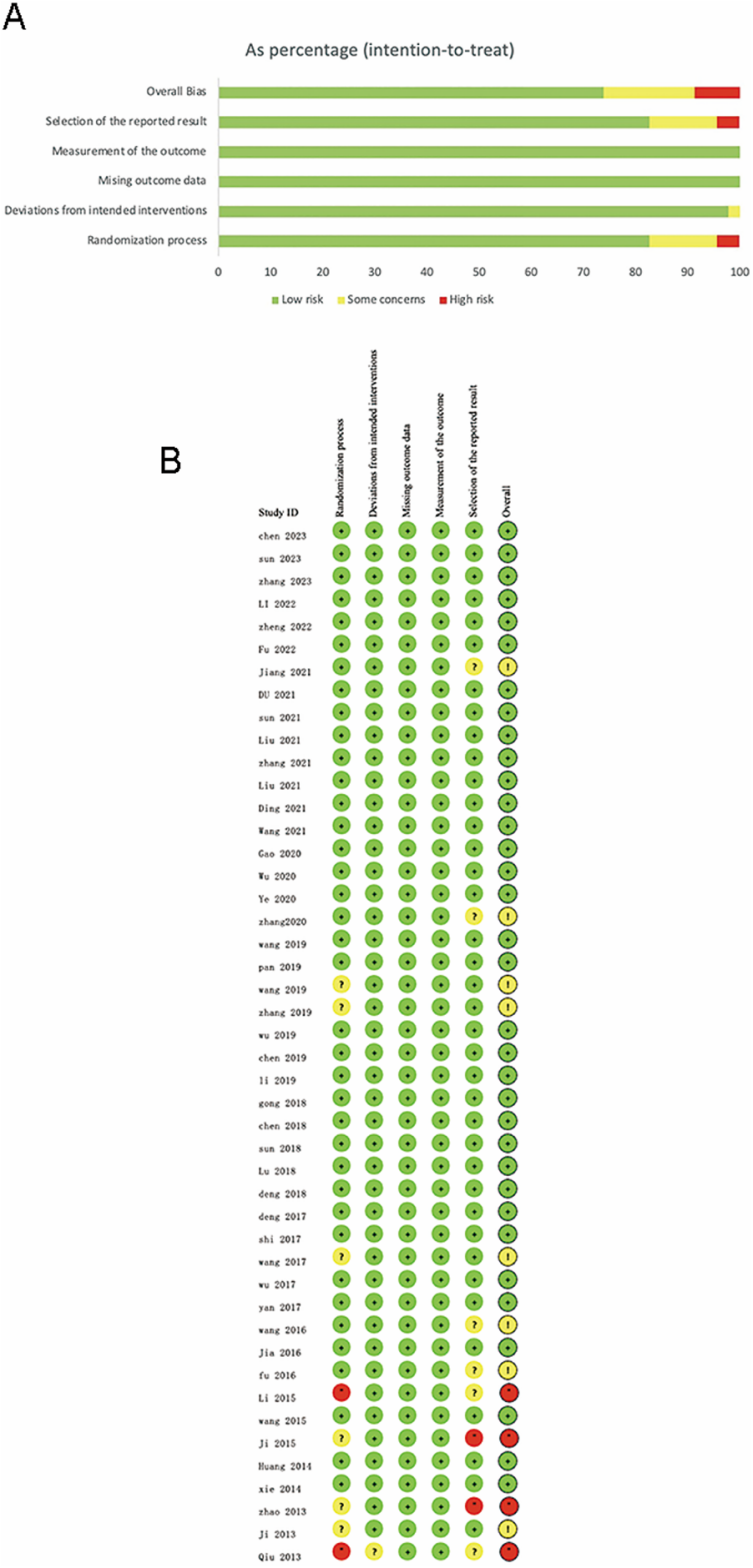
**(A)** Risk of bias graph. **(B)** Risk of bias graph.

4 RCTs were rated at high risk due to unknown or incorrect randomization methods and missing multiple efficacy indicators. 8 RCTs were rated as some concerns, with 4 RCTs not describing randomization methods and 4 RCTs missing multiple efficacy indicators. The remaining 34 RCTs were rated at low risk of bias (Figure 2).

### 3.4 NMA

#### 3.4.1 Main outcome indicators

##### 3.4.1.1 Number of angina attacks

**Figure 3:**
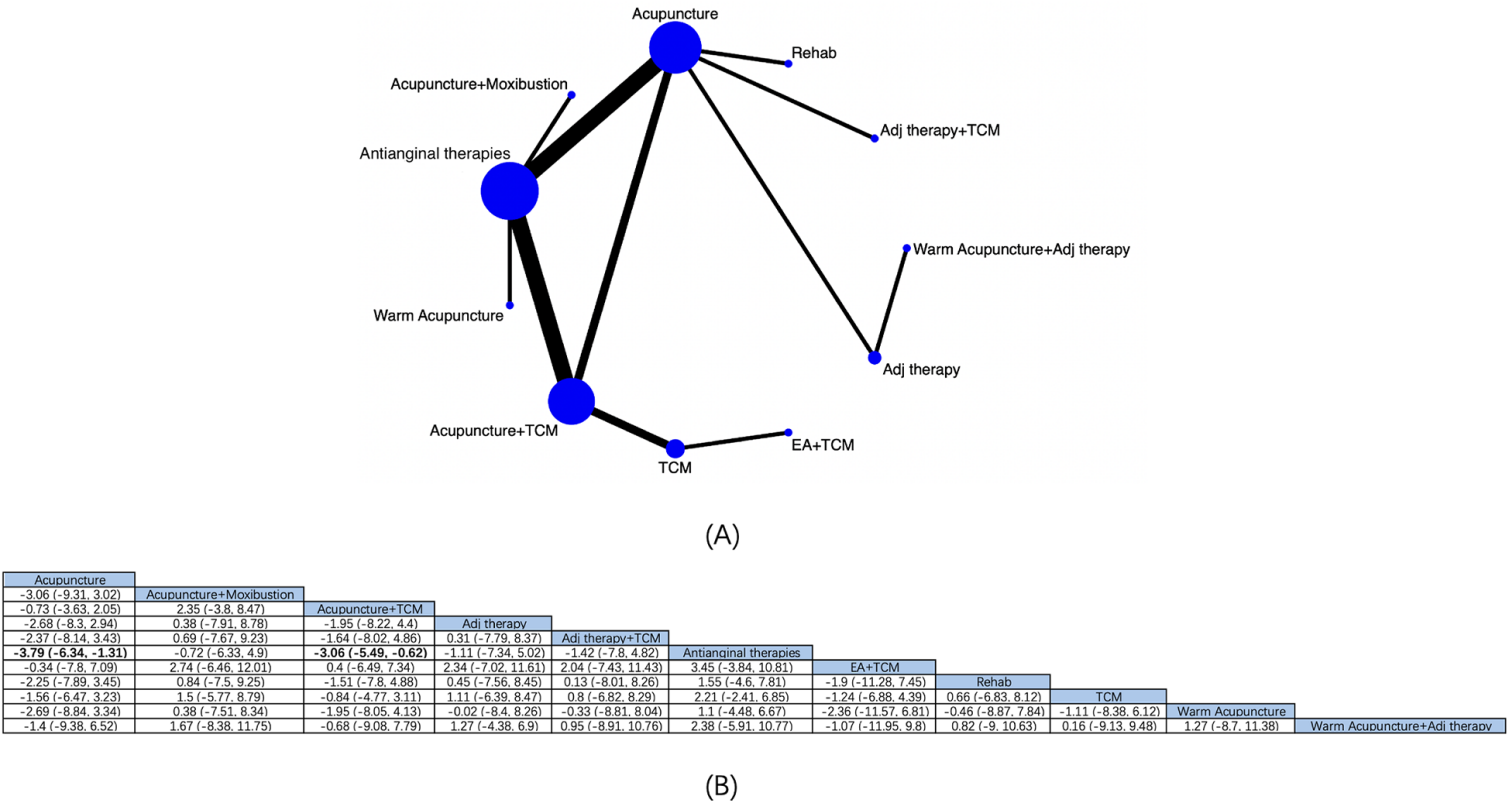
Network diagrams and NMA results. **(A)** Network plot of the number of angina episodes. **(B)** Relative impact of different interventions on the number of angina episodes. Estimates are depicted as MD and 95% CrI. Comparisons are interpreted by Reading from left to right. The estimates of treatment effects can be found at the point where the specified column intervention intersects with the specified row intervention. Noteworthy findings are highlighted in bold text.

11 intervention modalities for angina pectoris from 19 RCTs were comprehensively assessed for their effectiveness in reducing angina episodes (Figure 3A). NMA results manifested that (Figure 3B) compared with antianginal therapies, based on antianginal therapies, acupuncture [MD: −3.79; 95% CrI (−6.34, −1.31)] and acupuncture + TCM [MD: −3.06; 95% CrI (−5.49, −0.62)] had reduced the number of angina episodes, with marked differences. Based on the SUCRA score, acupuncture was the most effective treatment (SUCRA score: 78.2%) (Supplementary Figure S1; Supplementary Table S5).

##### 3.4.1.2 Duration of angina attacks

**Figure 4:**
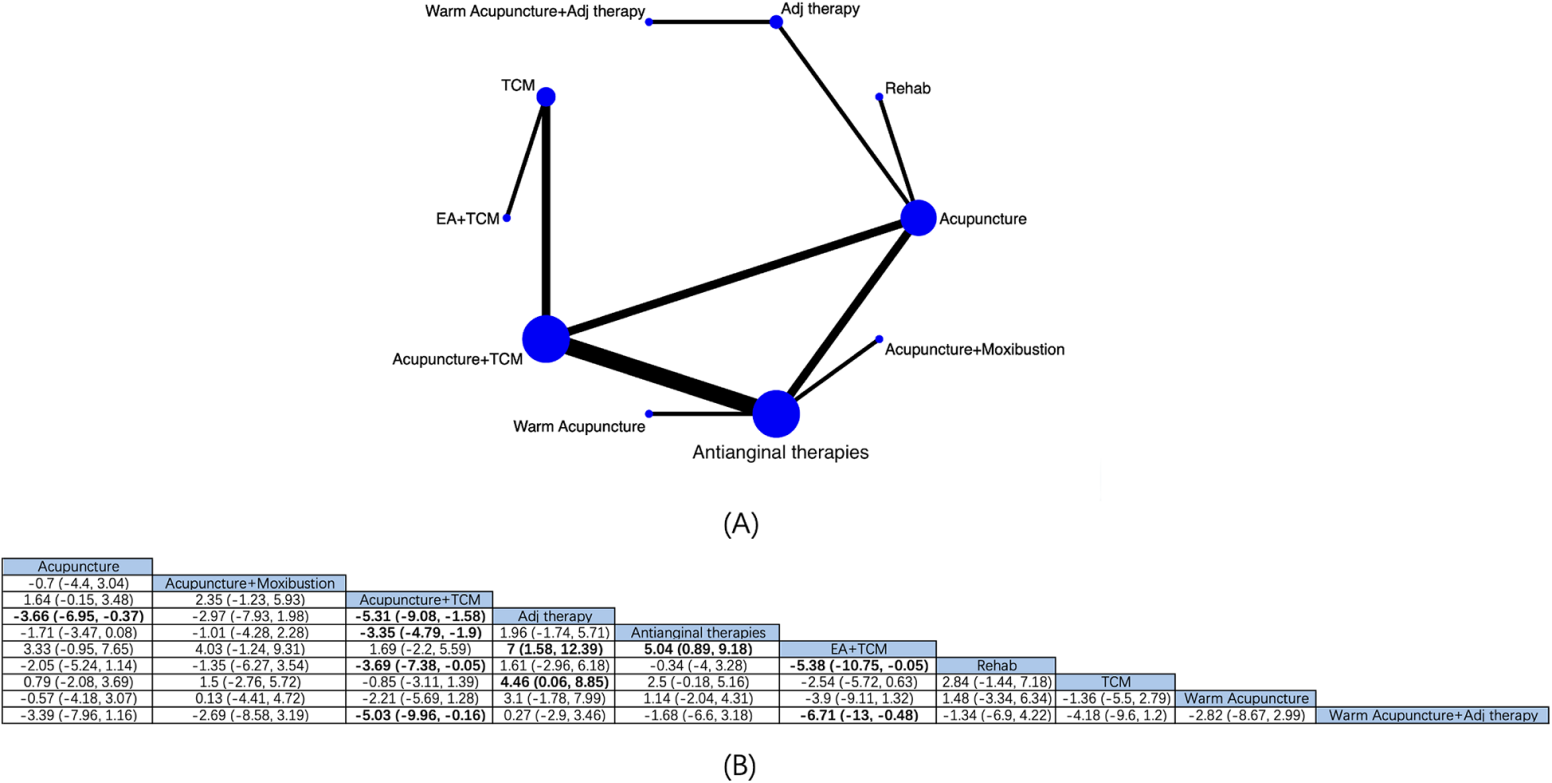
Network diagrams and NMA results. **(A)** Network plot of the duration of angina attacks. **(B)** Relative impact of different interventions on the duration of angina attacks. Estimates are depicted as MD and 95% CrI. Comparisons are interpreted by Reading from left to right. The estimates of treatment effects can be found at the point where the specified column intervention intersects with the specified row intervention. Noteworthy findings are highlighted in bold text.

10 interventions for angina pectoris from 16 RCTs were comprehensively assessed for their effectiveness in reducing the duration of angina attacks (Figure 4A). NMA results manifested that (Figure 4B) compared with antianginal therapies, based on antianginal therapies, acupuncture + TCM [MD: −3.35; 95% CrI (−4.79, −1.9)] and EA + TCM [MD −5.04; 95% CrI (−9.18, −0.89)] had reduced patients’ anginal attack durations, and EA + TCM was superior to Adj therapy [MD: 7; 95% CrI (1.58, 12.39)], rehabilitation [MD: −5.38; 95% CrI (−10.75, −0.05)], and warm acupuncture + Adj therapy [MD: −6.71; 95% CrI (−13, −0.48)], with substantial differences. Based on the SUCRA score, EA + TCM was the most effective (SUCRA score: 95.1%) (Supplementary Figure S2; Supplementary Table S6).

#### 3.4.2 Secondary outcome indicators

##### 3.4.2.1 Clinical efficacy

**Figure 5:**
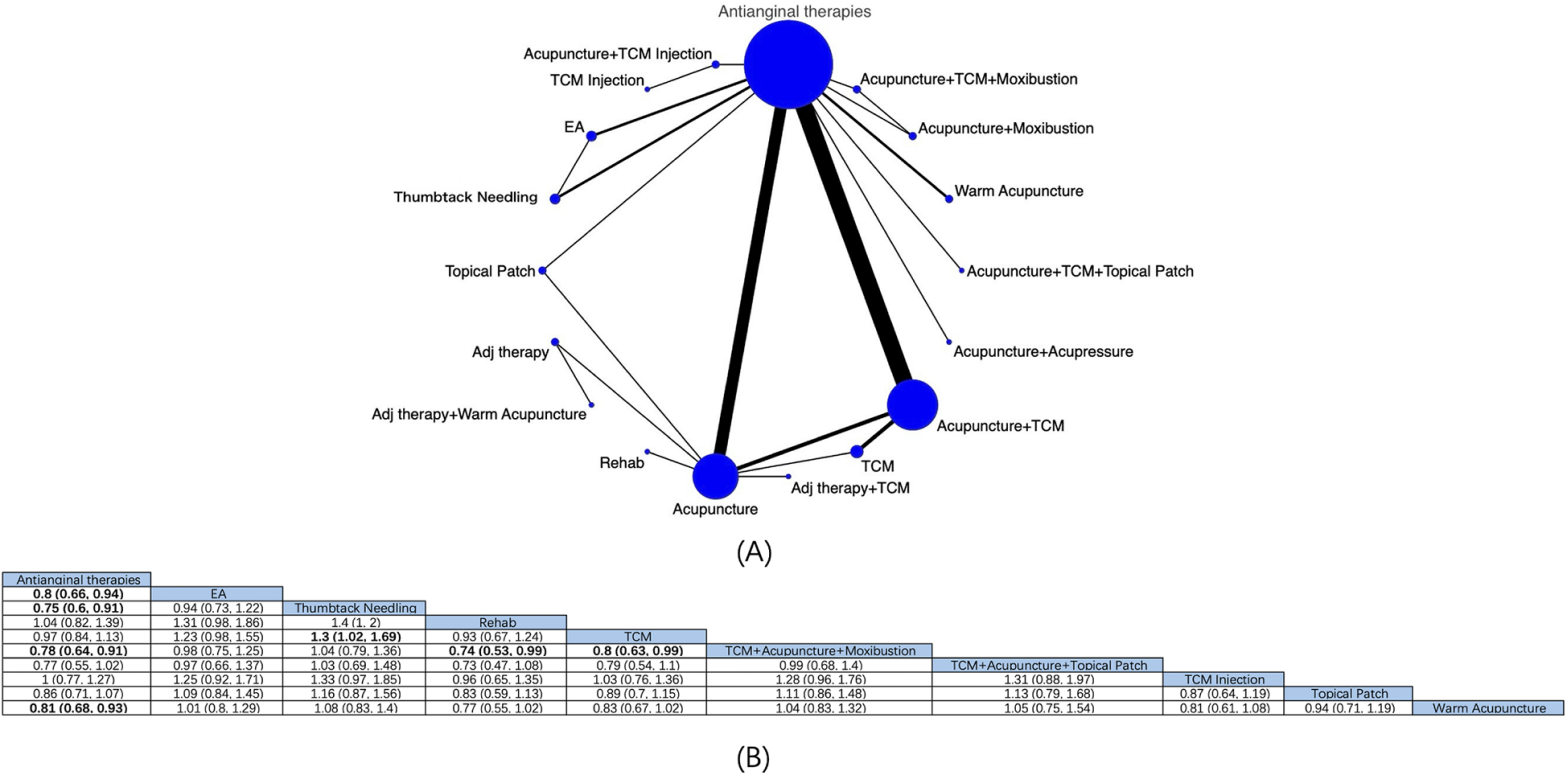
Network diagrams and NMA results. **(A)** Network diagram of clinical efficacy. **(B)** Relative impact of different intervention modalities on patients’ clinical efficacy. Estimates are depicted as RR and 95% CrI. Comparisons are interpreted by Reading from left to right. The estimates of treatment effects can be found at the point where the specified column intervention intersects with the specified row intervention. Noteworthy findings are highlighted in bold text. located at the intersection of the defined column intervention and the defined row intervention. Significant results are presented in bold.

The effectiveness of 18 interventions for angina pectoris in clinical symptom improvement was assessed by a pooled analysis of 41 RCTs (Figure 5A). NMA showed (Figure 5B) that based on antianginal therapies, thumbtack needling [RR: 0.75; 95% CrI (0.6, 0.91)], EA [RR: 0.8; 95% CrI (0.66, 0.94)], acupuncture + TCM + moxibustion [RR: 0.78; 95% CrI (0.64, 0.91)], and warm acupuncture [RR: 0.81; 95% CrI (0.68, 0.93)] were more effective than antianginal therapies in alleviating angina clinical symptoms. Thumbtack needling was superior to TCM [RR: 1.3; 95% CrI (1.02, 1.69)], and TCM + acupuncture was superior to TCM [RR: 0.8; 95% CrI (0.63,0.99)] and rehabilitation [RR: 0.74; 95% CrI (0.53,0.99)], with statistically notable differences. Based on the SUCRA score, thumbtack needling was considered the most effective modality (SUCRA score: 82.1%) (Supplementary Figure S3; Supplementary Table S7).

##### 3.4.2.2 ECG efficacy

**Figure 6:**
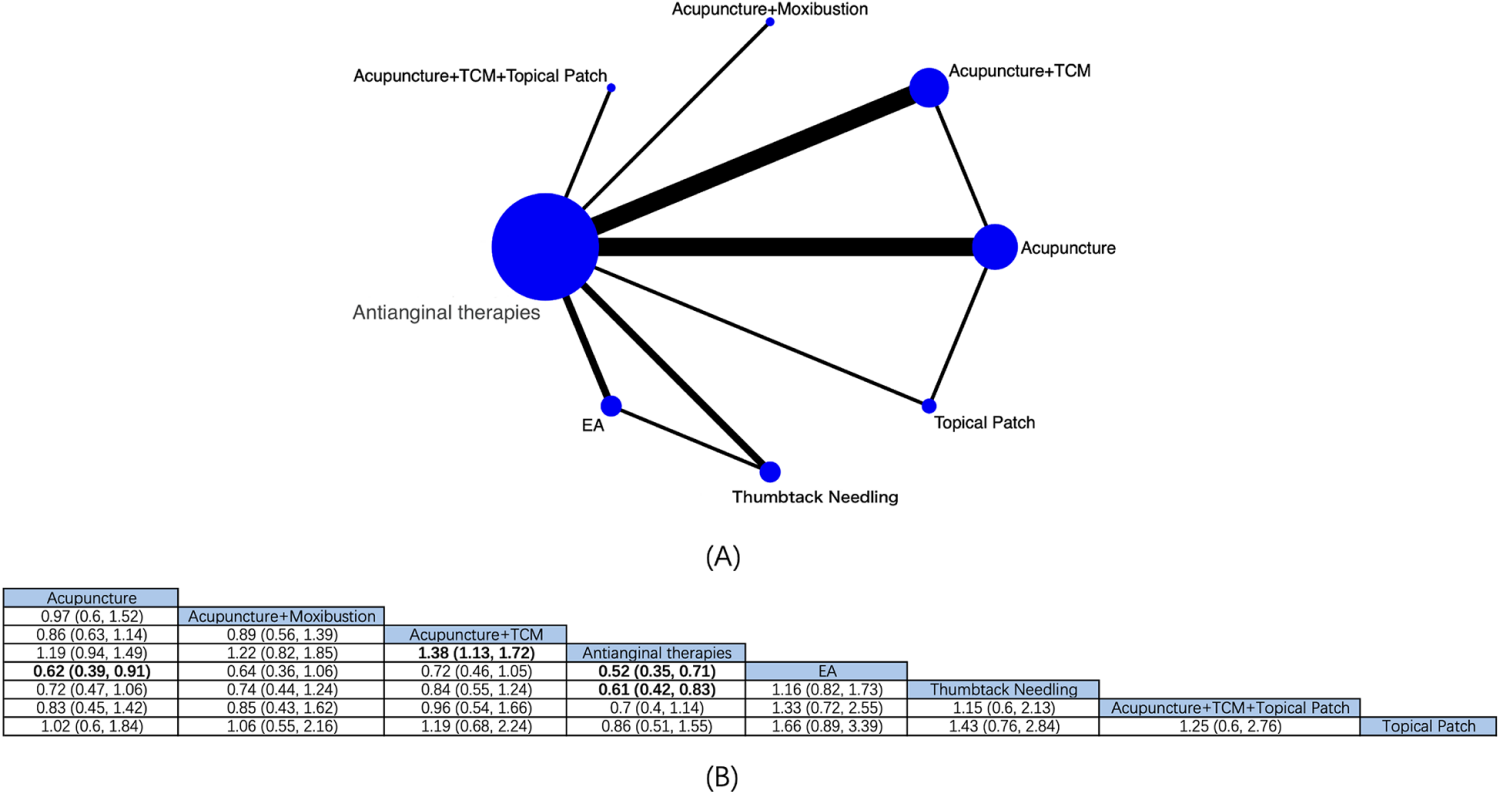
Network diagrams and NMA results. **(A)** Network plot of ECG efficiency. **(B)** Relative impact of different intervention modalities on the effective rate of ECG improvement. Estimates are depicted as RR and 95% CrI. Comparisons are interpreted by Reading from left to right. The estimates of treatment effects can be found at the point where the specified column intervention intersects with the specified row intervention. Noteworthy findings are highlighted in bold text.

8 intervention modalities for angina pectoris from 14 RCTs were comprehensively assessed for their effectiveness in ECG improvement (Figure 6A). NMA showed (Figure 6B) that based on antianginal therapies, EA [RR: 0.52; 95% CrI (0.35, 0.71)], acupuncture + TCM [RR: 1.38; 95% CrI (1.13, 1.72)], and thumbtack needling [RR 0.61; 95% CrI (0.42, 0.83)] boosted the effective rate of ECG improvement in patients with angina pectoris, and EA was superior to acupuncture [RR: 0.62; 95% CrI (0.39, 0.91)], with statistical differences. Based on the SUCRA score, EA was considered the best treatment modality (SUCRA score: 92.9%) (Supplementary Figure S4; Supplementary Table S8).

##### 3.4.2.3 TCM symptom score

6 interventions from 11 RCTs were comprehensively assessed (Supplementary Figure S5). Based on antianginal therapies, acupuncture + TCM [MD: −0.82; 95% CrI (−1.44, −0.22)] was superior to antianginal therapies in improving TCM symptom scores, with substantial differences. Based on the SUCRA score, acupuncture + TCM + topical patch was the most effective treatment (SUCRA score: 75.0%) (Supplementary Figure S6; Supplementary Table S9).

##### 3.4.2.4 Nitroglycerin use

5 intervention modalities from 8 RCTs were comprehensively assessed (Supplementary Figure S7). Based on antianginal therapies, compared with Adj therapy, acupuncture [MD: −13.8; 95%C CrI (−27.46, −0.1)] and acupuncture + TCM [MD −16.87; 95% CrI (−33.32, −0.44)] had reduced patients’ nitroglycerin use, with statistical differences. Based on the SUCRA score, acupuncture + TCM was the most effective treatment (SUCRA score: 87.9%) (Supplementary Figure S8; Supplementary Table S10).

### 3.5 Adverse events

5 interventions from the 46 RCTs included in this study reported adverse events associated with acupuncture therapies. One trial documented six cases of transient headache, dizziness, and palpitations (45). Another trial reported two cases each of gastrointestinal discomfort, liver function abnormalities, and local bleeding in the acupuncture group (54). Minor local reactions, such as bleeding at needle sites and adhesive allergies, were observed in one trial each (15). Additionally, one trial recorded two cases of vomiting and one case of fatigue in the acupuncture group (41), while another reported one case of nausea (53).

### 3.6 Publication bias

All outcome indicators were tested for publication bias (Supplementary Figure S9). According to the funnel plots, the number of angina attacks, duration of angina attacks, and clinical efficacy showed good symmetry suggesting comprehensive findings on these indicators and no significant publication bias. However, the funnel plots showed certain asymmetry in ECG improvement, nitroglycerin use, and TCM symptom score, suggesting potential publication bias.

## 4 Discussion

In recent years, several clinical RCTs and meta-analyses have reported that acupuncture combined with different interventions can improve clinical symptoms and quality of life in angina patients (11, 14, 65). This NMA comprehensively evaluated acupuncture combined with multiple interventions for angina pectoris based on antianginal therapies by analyzing the most recent data from the past 10 years, including acupuncture, thumbtack needling, EA, acupuncture + TCM + moxibustion, and warm acupuncture. 46 RCTs were analyzed. The results noted that relative to antianginal therapies, these interventions demonstrated certain advantages in the number and duration of angina attacks, clinical efficacy, ECG efficacy, TCM symptom scores, and nitroglycerin use. Acupuncture alone was the most effective in reducing the frequency of angina attacks, EA + TCM was the most effective in reducing the duration of angina attacks, thumbtack needling was most significant in improving clinical symptoms of angina pectoris, and EA exhibited the best efficacy in improving ECG efficacy.

In the number of angina episodes, acupuncture + antianginal therapies was superior to antianginal therapies and was considered the optimal treatment. Nonetheless, an NMA by Li et al. concluded that acupuncture did not diminish the number of angina episodes (65). Discrepancy may be due to the heterogeneity of enrolled populations, including angina types, enrolled interventions, and the number of cases. However, the meta-analysis by Lu et al. concluded that acupuncture + antianginal therapies diminished the number of angina attacks relative to antianginal therapies alone (13), consistent with our findings. Acupuncture is one of the oldest treatment modalities in China. In reducing angina episodes, its therapeutic mechanism may involve the regulation of sympathetic nerves and the improvement of vascular endothelial function. Acupuncture regulates the activity of the sympathetic nervous system by stimulating specific acupoints, thereby reducing myocardial oxygen consumption and relieving angina symptoms. Additionally, acupuncture can improve vascular endothelial function, promote vasodilatation, and increase the blood flow of coronary arteries, thus improving myocardial blood supply and reducing angina episodes (66, 67). These mechanisms may collaborate to contribute to its significant clinical efficacy in decreasing the frequency of angina attacks.

In terms of the duration of angina attacks, EA was most effective based on antianginal therapies, while EA + TCM was the most effective in terms of electrocardiographic efficacy. EA is thought to improve myocardial function through various mechanisms. For example, by modulating the expression of energy metabolism-related ATP synthase F(0) complex subunit G1, EA enhances energy synthesis in myocardial ischemic regions. Meanwhile, by regulating the inflammation-related TLR4/MyD88/NF-κB pathway, EA could reduce the release of inflammatory cytokines, thereby repressing inflammatory responses. EA can also reduce oxidative stress-induced myocardial injury by activating the endogenous antioxidant pathway, increasing superoxide dismutase, and decreasing malondialdehyde production. In addition, EA can prevent cardiac sympathetic activation (68–70). Although the potential efficacy of acupuncture for ECG improvement has also been reported in systematic reviews (12), suggesting that acupuncture may improve myocardial blood supply or modulate autonomic function to some extent. However, the quality of evidence from these studies is generally low. Moreover, the specific mechanism of acupuncture in improving ECG, especially in terms of ST-segment elevation and T-wave inversion improvement, is not clear. Although this study discusses the role of acupuncture on ECG improvement based on the existing literature and physiological mechanisms, these findings still need to be validated by further high-quality RCTs.

In terms of improvement in clinical symptoms of angina, the SUCRA score found that thumbtack needling performed most prominently based on antianginal therapies and significantly better than antianginal therapies and TCM. Its efficacy in treating angina pectoris may be related to its ability to improve myocardial ischemia by prolonged stimulation of acupoints and modulation of vascular growth factor expression (71). Continuous stimulation of thumbtack needles promotes the release of vascular endothelial growth factor and other angiogenesis-related factors, which are crucial in improving myocardial blood flow and reducing ischemic areas. In addition, the weak stimulation of thumbtack needles may relieve angina symptoms by reducing the inflammatory response through modulation of the neuroendocrine system (12, 72). Combined with these mechanisms, thumbtack needling not only excels in symptomatic relief but may also improve cardiac function through multiple physiologic pathways. The safety, simplicity, cost-effectiveness, and clinical applicability of thumbtack needles call for further research and clinical trials to understand its specific mechanisms and long-term effects on angina pectoris.

Acupuncture + TCM was notably better than antianginal therapies in improving TCM symptom scores (mainly including chest pain, chest tightness, palpitations, and fatigue). The difference was not statistically notable between acupuncture + TCM + topical patch and antianginal therapies in TCM symptom improvement, which may be due to the small sample size of acupuncture + TCM + topical patch, resulting in insufficient statistical efficacy. However, the SUCRA score illustrated that acupuncture + TCM + topical patch was the most effective treatment. In the future, statistical efficacy can be improved by including the latest studies to obtain more reliable results in TCM symptom scores in angina patients with acupuncture modalities.

There was no statistical significance in the reduction of nitroglycerin use with acupuncture, TCM, and acupuncture + TCM compared with antianginal therapies, inconsistent with the findings of Liu et al.'s meta-analysis that acupuncture + TCM can reduce nitroglycerin use in patients with angina pectoris (73). This may be related to the methodology used and the heterogeneity of the included population, as our study included cases with multiple treatment modalities. We assessed the effects of all interventions by NMA indirect comparisons (i.e., by comparison of a common control group) and evinced that acupuncture + TCM was the optimal treatment based on SUCRA scores. Combined with the study of Liu et al, we concluded that acupuncture + TCM is advantageous in reducing nitroglycerin use in patients with angina pectoris. Rigorous RCTs are warranted in the future to demonstrate the evidence-based rationale for acupuncture + TCM in reducing nitroglycerin use in patients with angina pectoris.

In terms of safety, only five studies reported a small number of adverse effects, including transient headache, nausea, and localized reactions, and serious adverse effects were not reported. The current results suggest that acupuncture combined with different therapies not only demonstrates significant efficacy based on the antianginal therapy but also has the advantage of high safety and few adverse effects.

Clinical decision-making for acupuncture-related therapies should be based on the patient's specific condition, personal wishes, and the characteristics of angina symptoms. For example, in patients with a high number of angina episodes, acupuncture therapy may provide significant benefits. In the context of precision and individualized medicine, different acupuncture therapies may further improve patient symptoms and enhance quality of life.

## 5 Strengths and limitations

To our knowledge, this is the first NMA to compare the effects of acupuncture combined with multiple interventions on various outcomes in angina pectoris and to rank all interventions based on these outcomes. This study provides evidence for optimizing acupuncture-based interventions for angina pectoris. However, several limitations of this NMA should be acknowledged. First, some included studies did not clearly specify diagnostic methods (e.g., stress tests, coronary CT, or coronary angiography) and only stated that the diagnosis was based on textbooks or guidelines. The lack of clear diagnostic descriptions may have led to inconsistencies in the diagnostic standards of the study populations. Future studies should adopt clear and standardized diagnostic methods to enhance the reliability of meta-analyses. Second, the limited sample size of non-ischemic angina patients and the lack of data on revascularization rates and outcomes prevented a comprehensive analysis of treatment differences between ischemic and non-ischemic patients. Although this study primarily focuses on symptom management, the potential impact of revascularization cannot be fully excluded. Future research should explicitly document these variables for more comprehensive evaluations. However, of the 46 studies eligible for inclusion, only 19 reported the relatively internationally recognized frequency of angina attacks as an efficacy indicator, while 41 used clinical outcome improvement. Variations in the efficacy criteria used in these studies were addressed using random-effects models and consistency tests to reduce the impact of heterogeneity. Although these criteria are based on official guidelines and integrate TCM perspectives, differences in efficacy assessments and subjective measures may limit the international recognition of acupuncture in angina treatment. Future studies should adopt internationally recognized standards, such as the frequency of angina attacks, exercise tolerance tests (ETT), Seattle Angina Questionnaire (SAQ) scores, and Canadian Cardiovascular Society (CCS) classification (6, 7), while incorporating clear and standardized diagnostic criteria along with the unique efficacy evaluation systems of TCM to enhance global acceptance. Lastly, the studies analyzed in this research were primarily published in Chinese medical journals, which may lead to regional bias and limit the generalizability of the results. Further high-quality randomized controlled trials are needed to validate our findings.

## 6 Conclusions

No single intervention was effective for all indices in angina patients. Based on antianginal therapy in all patients, acupuncture showed the best efficacy in reducing the number of angina attacks, and EA + TCM was the most effective modality in shortening the duration of angina attacks. In addition, thumbtack needling was the most significant in improving the clinical symptoms of angina pectoris, and EA had the best efficacy in improving the electrocardiogram. In terms of TCM symptom score, acupuncture + TCM + topical patch was considered the optimal treatment modality. In terms of reducing nitroglycerin use, acupuncture + TCM showed the highest effectiveness. These results suggest that acupuncture and its combination therapies have significant clinical applications in angina pectoris. Due to some limitations of current clinical data, future research should prioritize larger sample sizes, longer follow-up duration, and more rigorous study designs to validate these findings.

## Data Availability

The original contributions presented in the study are included in the article/Supplementary Material, further inquiries can be directed to the corresponding author.
