# Supplementary material for: Acupuncture combined with multiple therapies for angina pectoris: a systematic review and network meta-analysis
Source: Front Cardiovasc Med. 2025 Jan 30;12:1463170. doi: 10.3389/fcvm.2025.1463170 (PMC11841414; doi:10.3389/fcvm.2025.1463170)
Supplement: Supplementary file 1 [file Datasheet1.docx]

**Supplementary materials**

**Supplementary Table**

**Table S1 Searching Strategies.**

**Pubmed: 183 records**

**#1** "Angina Pectoris"[Mesh] OR "Angina, Stable"[Mesh] OR "Angina, Unstable"[Mesh]

**#2** Angina Pectoris OR Stenocardia OR Stenocardias OR Angor Pectoris OR Angina, Stable OR Stable Angina OR Stable Anginas OR Chronic Stable Angina OR Chronic Stable Anginas OR Angina Pectoris, Stable OR Angina Pectori, Stable OR Stable Angina Pectori OR Stable Angina Pectoris OR Angina, Unstable OR Unstable Anginas OR Angina Pectoris, Unstable OR Angina Pectori, Unstable OR Unstable Angina OR Angina at Rest OR Preinfarction Angina OR Preinfarction Anginas OR Myocardial Preinfarction Syndrome OR Myocardial Preinfarction Syndromes OR Angina OR anginal attack OR effort angina pectoris ：[Title/Abstract]

**#3** #1 OR #2

**#4** ("Acupuncture"[Mesh] OR "Acupuncture Therapy"[Mesh] OR "Acupuncture, Ear"[Mesh] OR "Acupuncture Points"[Mesh]) OR "Electroacupuncture"[Mesh]

**#5** Acupuncture OR Pharmacopuncture OR shonishin OR acupuncture treatment OR needling OR needle OR acupuncture therapy OR Acupuncture Treatment OR Acupuncture Treatments OR Pharmacoacupuncture Treatment OR Pharmacoacupuncture Therapy OR Acupotomy OR Acupotomies OR acupressure-acupuncture therapy OR Acupuncture, Ear OR Ear Acupunctures OR Auricular Acupuncture OR Ear Acupuncture OR Auricular Acupunctures OR auricular acupuncture OR auriculo-acupuncture OR auriculoacupuncture OR auriculotherapy OR earlobe acupuncture OR auricular needle OR electroacupuncture OR electric acupuncture OR electrical acupoint stimulation OR electrical acupuncture OR electro-acupuncture OR electrode acupuncture OR electronic acupuncture OR acupuncture points OR Acupuncture Point OR Acupoints OR Acupoint OR acu-point OR Scalp acupuncture OR Ocular acupuncture OR Auriculotherapy OR electrical acupoint stimulation：[Title/Abstract]

**#6** #4 OR #5

**#7** #3 AND #6

**Cochrane Library: 113 records**

**#1** MeSH descriptor: [Angina Pectoris] explode all trees

**#2** MeSH descriptor: [Angina, Stable] explode all trees

**#3** MeSH descriptor: [Angina, Unstable] explode all trees

**#4** Angina Pectoris OR Stenocardia OR Stenocardias OR Angor Pectoris OR Angina, Stable OR Stable Angina OR Stable Anginas OR Chronic Stable Angina OR Chronic Stable Anginas OR Angina Pectoris, Stable OR Angina Pectori, Stable OR Stable Angina Pectori OR Stable Angina Pectoris OR Angina, Unstable OR Unstable Anginas OR Angina Pectoris, Unstable OR Angina Pectori, Unstable OR Unstable Angina OR Angina at Rest OR Preinfarction Angina OR Preinfarction Anginas OR Myocardial Preinfarction Syndrome OR Myocardial Preinfarction Syndromes OR Angina OR anginal attack OR effort angina pectoris：ti,ab,kw

**#5** #1 OR #2 OR #3 OR #4

**#6** MeSH descriptor: [Acupuncture] explode all trees

**#7** MeSH descriptor: [Acupuncture Therapy] explode all trees

**#8** MeSH descriptor: [Acupuncture, Ear] explode all trees

**#9** MeSH descriptor: [Electroacupuncture] explode all trees

**#10** MeSH descriptor: [Acupuncture Points] explode all trees

**#11** Acupuncture OR Pharmacopuncture OR shonishin OR acupuncture treatment OR needling OR needle OR acupuncture therapy OR Acupuncture Treatment OR Acupuncture Treatments OR Pharmacoacupuncture Treatment OR Pharmacoacupuncture Therapy OR Acupotomy OR Acupotomies OR acupressure-acupuncture therapy OR Acupuncture, Ear OR Ear Acupunctures OR Auricular Acupuncture OR Ear Acupuncture OR Auricular Acupunctures OR auricular acupuncture OR auriculo-acupuncture OR auriculoacupuncture OR auriculotherapy OR earlobe acupuncture OR auricular needle OR electroacupuncture OR electric acupuncture OR electrical acupoint stimulation OR electrical acupuncture OR electro-acupuncture OR electrode acupuncture OR electronic acupuncture OR acupuncture points OR Acupuncture Point OR Acupoints OR Acupoint OR acu-point OR Scalp acupuncture OR Ocular acupuncture OR Auriculotherapy OR electrical acupoint stimulation：ti,ab,kw

**#12** #6 OR #7 OR #8 OR #9 OR #10 OR #11

**#13** #5 AND #12

**Web of Science: 186 records**

TS = (Angina Pectoris OR Stenocardia OR Stenocardias OR Angor Pectoris OR Angina, Stable OR Stable Angina OR Stable Anginas OR Chronic Stable Angina OR Chronic Stable Anginas OR Angina Pectoris, Stable OR Angina Pectori, Stable OR Stable Angina Pectori OR Stable Angina Pectoris OR Angina, Unstable OR Unstable Anginas OR Angina Pectoris, Unstable OR Angina Pectori, Unstable OR Unstable Angina OR Angina at Rest OR Preinfarction Angina OR Preinfarction Anginas OR Myocardial Preinfarction Syndrome OR Myocardial Preinfarction Syndromes OR Angina OR anginal attack OR effort angina pectoris) AND TS = (Acupuncture OR Pharmacopuncture OR shonishin OR acupuncture treatment OR needling OR needle OR acupuncture therapy OR Acupuncture Treatment OR Acupuncture Treatments OR Pharmacoacupuncture Treatment OR Pharmacoacupuncture Therapy OR Acupotomy OR Acupotomies OR acupressure-acupuncture therapy OR Acupuncture, Ear OR Ear Acupunctures OR Auricular Acupuncture OR Ear Acupuncture OR Auricular Acupunctures OR auricular acupuncture OR auriculo-acupuncture OR auriculoacupuncture OR auriculotherapy OR earlobe acupuncture OR auricular needle OR electroacupuncture OR electric acupuncture OR electrical acupoint stimulation OR electrical acupuncture OR electro-acupuncture OR electrode acupuncture OR electronic acupuncture OR acupuncture points OR Acupuncture Point OR Acupoints OR Acupoint OR acu-point OR Scalp acupuncture OR Ocular acupuncture OR Auriculotherapy OR electrical acupoint stimulation)

**Embase: 462 records**

**#1** 'angina pectoris'/exp OR 'stable angina pectoris'/exp OR 'unstable angina pectoris'/exp

#2 'angina pectoris':ab,ti OR stenocardia:ab,ti OR stenocardias:ab,ti OR 'angor pectoris':ab,ti OR 'angina, stable':ab,ti OR 'stable angina':ab,ti OR 'stable anginas':ab,ti OR 'chronic stable angina':ab,ti OR 'chronic stable anginas':ab,ti OR 'angina pectoris, stable':ab,ti OR 'angina pectori, stable':ab,ti OR 'stable angina pectori':ab,ti OR 'stable angina pectoris':ab,ti OR 'angina, unstable':ab,ti OR 'unstable anginas':ab,ti OR 'angina pectoris, unstable':ab,ti OR 'angina pectori, unstable':ab,ti OR 'unstable angina':ab,ti OR 'angina at rest':ab,ti OR 'preinfarction angina':ab,ti OR 'preinfarction anginas':ab,ti OR 'myocardial preinfarction syndrome':ab,ti OR 'myocardial preinfarction syndromes':ab,ti OR angina:ab,ti OR 'anginal attack':ab,ti OR 'effort angina pectoris':ab,ti

**#3** #1 OR #2

**#4** 'acupuncture'/exp OR 'auricular acupuncture'/exp OR 'electroacupuncture'/exp OR 'acupuncture point'/exp

#5 acupuncture OR pharmacopuncture:ab,ti OR shonishin:ab,ti OR needling:ab,ti OR needle:ab,ti OR 'stable anginas':ab,ti OR 'acupuncture therapy':ab,ti OR 'acupuncture treatment':ab,ti OR 'acupuncture treatments':ab,ti OR 'pharmacoacupuncture treatment':ab,ti OR 'pharmacoacupuncture therapy':ab,ti OR acupotomy:ab,ti OR acupotomies:ab,ti OR 'acupressure-acupuncture therapy':ab,ti OR 'acupuncture, ear':ab,ti OR 'ear acupunctures':ab,ti OR 'auricular acupuncture':ab,ti OR 'ear acupuncture':ab,ti OR 'auricular acupunctures':ab,ti OR 'auriculo acupuncture':ab,ti OR auriculoacupuncture:ab,ti OR 'earlobe acupuncture':ab,ti OR 'auricular needle':ab,ti OR electroacupuncture:ab,ti OR 'electric acupuncture':ab,ti OR 'electrical acupuncture':ab,ti OR 'electro acupuncture':ab,ti OR 'electrode acupuncture':ab,ti OR 'electronic acupuncture':ab,ti OR 'acupuncture points':ab,ti OR 'acupuncture point':ab,ti OR acupoints:ab,ti OR acupoint:ab,ti OR 'acu point':ab,ti OR 'scalp acupuncture':ab,ti OR 'ocular acupuncture':ab,ti OR auriculotherapy:ab,ti OR 'electrical acupoint stimulation':ab,ti

#6 #4 OR #5

#7 #3 AND #6

**CNKI:277 records**

TKA = ('针灸'+'温针灸'+'电针'+'针刺'+'耳针'+'头针'+'头皮针'+'温针疗法') AND TKA = ('心绞痛'+'狭心症'+'心绞疼') AND TKA =（'随机'）

**VIP:203 records**

（针灸+温针灸+电针+针刺+耳针+头针+头皮针+温针疗法）

AND （心绞痛+狭心症+心绞疼）

AND （随机对照+随机+临床试验）

**Wanfang:297 records**

(针灸 or 温针灸 or 电针 or 针刺 or 耳针 or 头针 or 头皮针or 温针疗法)

AND (心绞痛 or 狭心症 or 心绞疼)

AND (随机)

**CBM:385 records**

**#1** "心绞痛"[常用字段:智能] OR "稳定型心绞痛"[常用字段:智能] OR "不稳定型心绞痛"[常用字段:智能] OR "心绞疼"[常用字段:智能] OR "狭心症"[常用字段:智能]

**#2**  "心绞痛"[不加权:扩展] OR "心绞痛, 不稳定型"[不加权:扩展] OR "心绞痛, 稳定型"[不加权:扩展]

**#3**  (#2) OR (#1)

#4 "针灸疗法"[不加权:扩展]

**#5** "针灸"[常用字段:智能] OR "温针灸"[常用字段:智能] OR "电针"[常用字段:智能] OR "针刺"[常用字段:智能] OR "耳针"[常用字段:智能] OR "头针"[常用字段:智能] OR "头皮针"[常用字段:智能] OR "温针疗法"[常用字段:智能]

**#6** (#5) OR (#4)

**#7** "随机对照"[常用字段:智能] OR "随机"[常用字段:智能]

**#8** (#7) AND (#6) AND (#3)

**Table S2 Clinical Symptom Improvement Rate Criteria**

| **Study** | **Efficacy Evaluation Criteria** |
| --- | --- |
| Zhang et al.(2023) | The efficacy evaluation criteria is formulated according to the "Guidelines for Clinical Research on New Traditional Chinese Medicines (Trial)": Markedly effective: The frequency, duration, and severity of angina attacks have significantly improved, with a reduction rate of HAMD-17 score ≥70%. Effective: The frequency, duration, and severity of angina attacks have improved, with a reduction rate of HAMD-17 score ≥30% and <70%. Ineffective: The frequency, duration, and severity of angina attacks have not significantly improved, or even worsened, with a reduction rate of HAMD-17 score <30%. |
| Sun et al.(2023) | Clinical efficacy evaluation criteria: Recent control: Clinical symptom score has improved by ≥90%. Markedly effective: Clinical symptom score has improved by ≥70%. Effective: Clinical symptom score has improved by ≥30% and <70%. Ineffective: Clinical symptom score has improved by <30% or symptoms have worsened. Total effective rate: Clinical total effective rate = Control rate + Markedly effective rate + Effective rate. |
| Chen et al.(2023) | The formulation is based on the efficacy criteria for angina pectoris in coronary heart disease outlined in the "Guidelines for Clinical Research on New Traditional Chinese Medicines (Trial)": Markedly effective: Angina symptoms have completely disappeared or been relieved by at least 2 levels compared to before treatment. Effective: Symptoms of angina have been relieved by 1 level compared to before treatment. Ineffective: There has been basically no difference in the angina symptoms compared to before treatment. Aggravated: Angina symptoms have worsened by at least 1 level compared to before treatment, or the frequency of angina attacks has increased, accompanied by greater pain severity and longer duration, among other indicators. |
| Fu et al.(2022) | Clinical efficacy evaluation criteria ("Guidelines for Clinical Research on Traditional Chinese Medicines (Trial)": Basic cure: Clinical symptoms have completely been resolved, with the electrocardiogram returning to normal and no further angina present. Markedly effective: The symptoms have mostly disappeared, the ST segment of the electrocardiogram has slightly decreased compared to before treatment but has not yet returned to normal, angina attacks have nearly ceased, and the frequency and number of angina attacks have significantly reduced. Effective: Symptoms have significantly improved, the ST segment of the electrocardiogram has decreased compared to before treatment, and the frequency and number of angina attacks have decreased. Ineffective: Symptoms have shown no improvement, the frequency and number of angina attacks have remained unchanged or worsened, and the electrocardiogram has shown no changes. Total effective rate: Total effective rate = (Basically cured + Markedly effective + Effective) / Total number of cases × 100%. |
| Li et al.(2022) | Efficacy evaluation criteria (referencing the "Guidelines for Clinical Research on New Traditional Chinese Medicines"): Markedly effective: Clinical symptoms of angina have disappeared, and the exercise stress test has turned negative. Effective: Clinical symptoms of angina have significantly improved, and the number of angina attacks has decreased. Ineffective: Angina symptoms have not improved or have worsened. |
| Zheng et al.(2022) | Efficacy evaluation criteria (referencing the "Guidelines for Clinical Research on New Traditional Chinese Medicines"):  Markedly effective: The number of angina attacks has decreased by ≥80%, and the total symptom score reduction rate for blood stasis and obstruction syndrome is ≥70%. Effective: The number of angina attacks has decreased by 50% to 80%, and the total symptom score reduction rate for blood stasis and obstruction syndrome is ≥30% but <70%. Ineffective: The number of angina attacks has decreased by ≤50%, and the total symptom score reduction rate for blood stasis and obstruction syndrome is <30%.  Formula for calculating the total symptom score reduction rate for blood stasis and obstruction syndrome: Total score reduction rate = (Total score before treatment - Total score after treatment) / Total score before treatment × 100%.  Total effective rate: Total effective rate = Markedly effective rate + Effective rate. |
| Liu et al.(2021) | Efficacy criteria for markedly effective: Clinical symptoms have disappeared, and cardiac function has returned to normal. Effective: Clinical symptoms have significantly been alleviated, and cardiac function has clearly improved. Ineffective: Clinical symptoms have shown no improvement or have even worsened. |
| Du et al.(2021) | Clinical efficacy evaluation criteria: Markedly effective: The number and duration of angina attacks have decreased by more than 80% after treatment, and the electrocardiogram has shown significant improvement in ischemic manifestations. Effective: After treatment, the number and duration of angina attacks both have decreased by 50% to 80%, and the electrocardiogram has shown improvement in ischemic manifestations. Ineffective: The above effects were not achieved after treatment. Total effective rate: Total effective rate = (Number of markedly effective cases + Number of effective cases) / Total number of cases × 100%. |
| Zhang et al.(2021) | Clinical efficacy evaluation criteria: Markedly effective: Symptoms and signs have significantly alleviated, and the electrocardiogram has basically recovered to normal levels. Effective: Symptoms and signs have improved, with the ST segment decreasing and recovering by more than 0.05 mV. Ineffective: Symptoms and signs have shown little to no improvement, with no significant changes in the electrocardiogram before and after treatment. Total effective rate = [(Number of markedly effective cases + Number of effective cases) / Total number of cases] × 100%. |
| Ding et al.(2021) | Efficacy evaluation criteria (referencing the "Guidelines for Clinical Research on New Traditional Chinese Medicines (Trial)"): Markedly effective: Symptoms have mostly disappeared or significantly improved, with the CCS classification improving by 2 or more levels, and the Traditional Chinese Medicine symptom score reduced by ≥70%. Effective: Symptoms have been alleviated, with the CCS classification improving by 1 level, and the TCM symptom score reduced by ≥30% but less than 70%. Ineffective: No improvement in symptoms or CCS classification, with the TCM symptom score reduced by less than 30%. Aggravated: Symptoms have worsened, and the TCM symptom score has increased. |
| Wang et al.(2021) | The efficacy evaluation criteria is formulated according to the "Guidelines for Clinical Research on New Traditional Chinese Medicines (Trial)": Markedly effective: Improvement rate of TCM symptom score ≥70%. Effective: Improvement rate of TCM symptom score is 30% to <70%. Ineffective: Improvement rate of TCM symptom score <30%.  Calculation formula for the improvement rate of TCM symptom score: Improvement rate = [(Pre-treatment TCM symptom score - Post-treatment TCM symptom score) ÷ Pre-treatment TCM symptom score] × 100%. |
| Liu et al.(2021) | Efficacy criteria: Cured: Reduction rate of TCM symptom score ≥ 90%. Markedly effective: The reduction rate of TCM symptom score is ≥ 70% and < 90%. Effective: The reduction rate of TCM symptom score is ≥ 30% and < 70%. Ineffective: The reduction rate of TCM symptom score is ＜30%. |
| Sun et al.(2021) | Efficacy evaluation criteria: Markedly effective: Significant improvement in clinical symptoms and signs, with symptom score reduction ≥ 70%. Effective: The clinical symptoms and signs have improved, and the symptom score has decreased by 30% to less than 70%. Ineffective: No obvious improvement in clinical symptoms and signs, and even worsening compared to before, with a symptom score reduction of less than 30%. |
| Jiang et al.(2021) | The formulation is based on the "Guidelines for Clinical Research on New Traditional Chinese Medicines (Trial)": 1. Markedly effective: The patient's original clinical symptoms have disappeared, and the TCM symptom score has decreased by 70% or more. Fasting blood glucose (FBG) and 2-hour postprandial blood glucose (2hPG) have dropped to normal levels. Glycated hemoglobin (Hemoglobin A1c, HbA1c) has dropped to 6.5% or decreased by more than 30% compared to before treatment. 2. Effective: The patient's clinical symptoms have significantly improved, and the TCM symptom score has decreased by 30% to 70%. FBG and 2hPG have decreased by 20%. HbA1c has decreased by 10%. 3. Ineffective: The patient's clinical symptoms have not significantly improved, and the TCM symptom score has decreased by less than 30%. The levels of FBG, 2hPG, and HbA1c have decreased but did not meet the above-mentioned standards. |
| Wu et al.(2020) | According to the "Standards for Evaluating the Efficacy of Traditional Chinese Medicine Diagnoses", the efficacy evaluation criteria are as follows: Markedly effective: After treatment, the patient's symptoms of chest tightness, palpitations, and angina have improved. Effective: The frequency and severity of angina attacks have decreased, and the electrocardiogram indicators have improved. Ineffective: Symptoms such as chest tightness, palpitations, and angina have not been alleviated, and the ischemic symptoms shown on the electrocardiogram have not improved. |
| Gao et al.(2020) | Clinical efficacy evaluation criteria: Markedly effective: After treatment, the patient's angina attacks have decreased in both number and duration by 80% or more, and the electrocardiogram has returned to normal. Effective: After treatment, the number and duration of angina attacks in the patient have decreased by 50% to 80%, and the electrocardiogram has shown significant improvement. Ineffective: The number and duration of the patient's angina attacks after treatment have decreased by less than 50%, and there has been no change or worsening in the electrocardiogram. |
| Ye et al.(2020) | Based on the "Guidelines for Clinical Research on New Traditional Chinese Medicines": Cured: Symptoms and signs have shown significant improvement, and the TCM symptom score has decreased by ≥90%. Markedly effective: Symptoms and signs have improved, with a reduction in TCM symptom score of ≥70%. Effective: Symptoms and signs have improved, and the TCM symptom score has decreased by ≥30%. Ineffective: There has been no significant improvement or worsening of symptoms and signs, and the TCM symptom score has decreased by less than 30%. |
| Zhang et al.(2020) | Efficacy evaluation based on the "Guidelines for Clinical Research on New Traditional Chinese Medicines (2002)": Markedly Effective: Symptoms of chest tightness and chest pain have disappeared or significantly eased, the number of angina attacks and the use of nitroglycerin have decreased by more than 80%, and the resting electrocardiogram has returned to normal; Effective: Symptoms of chest tightness and chest pain have improved, the number of angina attacks and the use of nitroglycerin have decreased by 50% to 80%, and the resting electrocardiogram has shown some improvement; Ineffective: The patient's symptoms of chest tightness and chest pain have not improved and may even have worsened, the number of angina attacks and the use of nitroglycerin have decreased by less than 50%, and there has been no change in the resting electrocardiogram. Total effective rate = (Number of markedly effective cases + Number of effective cases) / Total number of cases × 100%. |
| Pan et al.(2019) | According to the "Diagnosis and Treatment Recommendations for Unstable Angina", the efficacy evaluation criteria are as follows: Markedly effective: The number of angina attacks has decreased by more than 95%, the resting electrocardiogram has returned to normal, the duration of angina attacks has significantly shortened, and the use of nitroglycerin has decreased by more than 80%. Effective: The number of angina attacks has decreased by 50% to 95%, the resting electrocardiogram is generally normal, the duration of angina attacks has shortened, and the use of nitroglycerin has decreased by 50% to 80%. Ineffective: The standards for "Markedly Effective" or "Effective" have not been met, or the symptoms have worsened compared to before treatment. |
| Wang et al.(2019) | Efficacy evaluation criteria (referencing the "Guidelines for Clinical Research on New Traditional Chinese Medicines"): Markedly effective: The patient's angina symptoms, such as chest pain, palpitations, and chest tightness, have disappeared, and the electrocardiogram shows no abnormalities or is generally normal. Effective: The patient's angina symptoms have been alleviated, and the electrocardiogram has improved compared to before treatment. Ineffective: The patient's angina symptoms have not changed, and there has been little to no change in the electrocardiogram. Aggravated: The patient's angina symptoms and electrocardiogram changes have become more severe compared to before treatment. |
| Wu et al.(2019) | Efficacy evaluation criteria: Markedly effective: After treatment, the number of angina attacks in the patient has decreased by ≥80%. Effective: After treatment, the number of angina attacks in the patient has decreased by 50% to 80%. Ineffective: There has been no change in the patient's angina attacks after treatment. Total effective rate: Total effective rate = Markedly effective rate + Effective rate. |
| Chen et al.(2019) | Efficacy evaluation criteria for angina pectoris in coronary heart disease (referencing the "Guidelines for Clinical Research on New Traditional Chinese Medicines"): Markedly effective: The angina grade has decreased by 2 levels. For those originally classified as grade I or II, the angina has mostly disappeared, and no angina occurs with physical activity exceeding the normal level of exertion. Effective: The grade of angina has decreased by 1 level, and normal physical activity does not induce angina. Ineffective: There has been no significant improvement in the angina symptoms or the improvement has not reached the expected level, and angina occurs even with exertion below the normal level of physical activity. |
| Chen et al.(2018) | The formulation is based on "Guidelines for Clinical Research on New Traditional Chinese Medicines". ① Efficacy criteria for angina symptoms: Markedly effective: Angina has disappeared or nearly disappeared. Effective: The number, severity, and duration of angina attacks have significantly decreased. Ineffective: The severity of symptoms has remained mostly unchanged or worsened. |
| Gong et al.(2018) | Efficacy evaluation of angina: Markedly Effective: Angina symptoms have nearly disappeared, and the number of occurrence has decreased by 3/4 compared to before treatment; Effective: The number of angina attacks has decreased by more than 1/2 but not more than 3/4 compared to before treatment; Ineffective: The number of angina attacks has shown little to no reduction, or may have even increased compared to before treatment. |
| Lu et al.(2018) | Efficacy evaluation criteria: Markedly effective: After three courses of treatment, angina is not triggered by the same level of exertion, or the number of angina attacks has decreased by more than 95%. The resting electrocardiogram is normal. In the occasional occurrence of angina, the use of nitroglycerin has decreased by more than 80%. Effective: After three courses of treatment, the angina symptoms have improved; the resting electrocardiogram has shown some improvement but has not reached normal range; the number of angina attacks has decreased by 50% to 80%, or the use of nitroglycerin has decreased by more than 50%. Ineffective: After treatment, there has been no reduction in the number or severity of angina attacks; the resting electrocardiogram shows no significant difference compared to before treatment; and the dosage of nitrate medications remains unchanged. |
| Sun et al.(2018) | The efficacy criteria are based on the relevant efficacy standards outlined in the "Guidelines for Clinical Research on New Traditional Chinese Medicines": Markedly effective: The number of angina attacks has decreased by more than 80%, the use of nitroglycerin has reduced by more than 80%, and the angina grade has decreased by 2 levels. Effective: The number of angina attacks and the dosage of nitroglycerin have decreased by 50% to 80%, and the grade of angina has decreased by 1 level. Ineffective: No significant improvement in clinical symptoms and signs compared to before treatment. Total effective rate: Total effective rate = (Number of markedly effective cases + Number of effective cases) / Total number of cases × 100%. |
| Deng et al.(2018) | The formulation is based on the clinical studies on the efficacy criteria of angina pectoris in coronary heart disease in the "Guidelines for Clinical Research on New Traditional Chinese Medicines": Markedly effective: Symptoms have disappeared or been alleviated by more than two levels. Effective: Symptoms have been alleviated by one level. Ineffective: Symptoms have not been alleviated. Aggravated: The frequency, severity, or duration of pain has worsened. |
| Shi et al.(2017) | According to the efficacy evaluation criteria for angina, the efficacy evaluation is as follows: Markedly effective: The angina classification has decreased by 2 levels; those originally at grade I or II have essentially no angina, and no angina occurs with physical activity exceeding the normal level of exertion. Effective: The angina grade has decreased by 1 level, with no angina occurring during normal physical activity. Ineffective: No improvement in angina symptoms or the improvement has not reached the expected level, and angina occurs even with exertion below the normal level of physical activity. |
| Wang et al.(2017) | Diagnostic criteria for evaluating the efficacy of angina pectoris in coronary heart disease outlined in the "Guidelines for Clinical Research on New Traditional Chinese Medicines (2002)". And the efficacy evaluation criteria for angina pectoris in coronary heart disease and arrhythmia from the 1979 "Symposium on Integrated Traditional and Western Medicine for Treating Angina Pectoris in Coronary Heart Disease and Arrhythmia". |
| Deng et al.(2017) | The efficacy evaluation criteria are based on the total score of TCM symptoms, referencing the "Guidelines for Clinical Research on New Traditional Chinese Medicines" for evaluating the overall efficacy of TCM symptoms, with the following standards: Markedly effective: Clinical symptoms and signs have significantly improved, and the efficacy index is ≥70%. Effective: Clinical symptoms and signs have improved, with an efficacy index of ≥30% but <70%. Ineffective: Clinical symptoms and signs have shown no significant improvement, with an efficacy index of <30%. Aggravated: No improvement or worsening of clinical symptoms and signs, with an increase in the efficacy index by more than 10%.  Efficacy index calculation formula: Efficacy index = (Total TCM symptom score before treatment - Total TCM symptom score after treatment) / Score before treatment × 100%. |
| Yan et al.(2017) | Clinical treatment efficacy evaluation criteria: Markedly effective: The patient's chest pain has disappeared or nearly disappeared after treatment. Effective: The patient's chest pain has significantly been alleviated after treatment. Ineffective: No improvement in the patient's chest pain symptoms after treatment, and even exacerbation. |
| Wu et al.(2017) | The efficacy evaluation criteria are formulated based on symptom efficacy index, referencing the "Guidelines for Clinical Research on New Traditional Chinese Medicines": Markedly effective: The efficacy index of TCM symptoms is ≥ 66.67%. Effective: The efficacy index of TCM symptoms is ≥ 33.33% and < 66.67%. Ineffective: The efficacy index of TCM symptoms is < 33.33%. |
| Jia et al.(2016) | According to the "Efficacy Evaluation Criteria for Angina Pectoris in Coronary Heart Disease and ECG", the treatment efficacy of angina is classified as follows: Markedly effective: Angina is not triggered by the same level of exertion, or the number of attacks has decreased. Effective: The number of angina attacks has decreased to less than half of the original number. Ineffective: The frequency, severity, and duration of angina attacks have worsened compared to before treatment, or the angina grade has increased. |
| Fu et al.(2016) | Efficacy evaluation criteria of improving clinical symptoms (referencing the "Standards for Diagnosis and Treatment of Angina in Traditional Chinese Medicine"): Markedly effective: The patient's angina attacks have disappeared or the number of attacks has decreased by ≥80%, while nitroglycerin usage has decreased by ≥80%, and the electrocardiogram shows that the ST segment and T wave have basically returned to normal. Effective: The number of angina attacks has decreased by ≥50% but is below 80%, and the electrocardiogram shows a near-normal recovery with a flattened ST segment at a low level and inverted T waves. Ineffective: The criteria for marked effectiveness or effectiveness have not been met, and the patient continues to experience relatively frequent chest tightness and angina symptoms, with the condition even progressing and worsening. Total effective rate: Total effective rate = (Number of markedly effective cases + Number of effective cases) / Total number of cases × 100%. |
| Wang et al.(2016) | The efficacy criteria reference the efficacy evaluation standards for chest stuffiness and pain in the "Standards for Evaluating the Efficacy of Traditional Chinese Medicine Diagnoses"[12]. The criteria are used to assess the clinical treatment efficacy: Markedly effective: The patient's clinical symptoms have disappeared, and the results of physical and chemical examinations have improved by more than 75% compared to before. Improved: The patient's clinical symptoms have lessened, and the results of physical and chemical examinations have improved by 50% to 75%. Ineffective: The patient's clinical symptoms and physical and chemical examination results have not improved to the standards of marked effectiveness or improvement. |
| Jin et al.(2015) | The formulation is based on the "Clinical Research Guiding Principles for New Traditional Chinese Medicines in Treating Chest Stuffiness (Angina Pectoris in Coronary Heart Disease)" and the "Efficacy Evaluation Criteria for Angina Pectoris in Coronary Heart Disease and ECG" from the 1979 Symposium on Integrated Traditional and Western Medicine for Treating Angina Pectoris in Coronary Heart Disease and Arrhythmia |
| Li et al.(2015) | Efficacy indicators: Effective: Symptoms, medication dosage, and number of angina attacks have significantly improved or reduced by more than half. Ineffective: No change in the number of angina attacks, symptoms, and medication dosage after treatment. Aggravated: The number, duration, and severity of angina attacks have all intensified, and medication dosage has increased. |
| Huang et al.(2014) | The efficacy evaluation criteria are formulated referencing the "Guidelines for Clinical Research on New Traditional Chinese Medicines". |
| Qiu et al.(2013) | Based on the efficacy evaluation criteria for "chest stuffiness" in the "Standards for Evaluating the Efficacy of Traditional Chinese Medicine Diagnoses", it has been summarized as follows: Cured: Symptoms have completely disappeared, and the electrocardiogram and all relevant laboratory test results have returned to normal. Improved: The symptoms have eased, the number of attacks has decreased, the interval between attacks has lengthened, and the laboratory test results have shown improvement. Not cured: No improvement in main symptoms, and no changes in electrocardiogram examination results. |
| Jin et al.(2013) | The formulation is based on the "Clinical Research Guiding Principles for New Traditional Chinese Medicines in Treating Chest Stuffiness (Angina Pectoris in Coronary Heart Disease)" and the "Efficacy Evaluation Criteria for Angina Pectoris in Coronary Heart Disease and ECG" from the 1979 Symposium on Integrated Traditional and Western Medicine for Treating Angina Pectoris in Coronary Heart Disease and Arrhythmia |
| Zhao et al.(2013) | The efficacy of TCM symptoms is evaluated according to the relevant standards in the "Guidelines for Clinical Research on New Traditional Chinese Medicines": Markedly effective: The clinical symptoms and signs have significantly improved, and the TCM symptom score has decreased by more than 70%. Effective: Both clinical symptoms and signs have improved, with a reduction in TCM symptom score greater than 30%. Ineffective: The clinical symptoms and signs have not improved significantly and may have worsened, with a decrease in TCM symptom score of less than 30%. Aggravated: Clinical symptoms and signs have all worsened, and the TCM symptom score has not decreased. |

**Table S3 ECG Improvement Rate Criteria**

| **Study** | **Efficacy Evaluation Criteria** |
| --- | --- |
| Chen et al.(2023) | The formulation is based on efficacy criteria of electrocardiograms in angina pectoris in coronary heart disease from the "Guidelines for Clinical Research on New Traditional Chinese Medicines (Trial)": Markedly effective: The electrocardiogram has returned to normal levels. Effective: After treatment, the ST segment remains depressed but has risen by more than 0.05 mV compared to the pre-treatment level; the T wave has changed from inverted to flattened or from flattened to upright. Ineffective: The electrocardiogram shows little change compared to pre-treatment. Aggravated: The ST segment has further decreased by more than 0.05 mV from its pre-treatment depressed level; the T wave has changed from upright to flattened or from flattened to inverted. |
| Jiang et al.(2021) | ECG efficacy evaluation criteria: Markedly effective: The electrocardiogram has returned to normal. Effective: The ST segment has decreased and then risen by more than 0.05 mV, but has not returned to normal levels; the inverted T waves in the main leads have become less pronounced; or the T wave has changed from flattened to upright. Ineffective: No significant improvement in electrocardiogram. |
| Wang et al.(2019) | Efficacy is evaluated using the relevant efficacy criteria from the "Expert Consensus on Traditional Chinese Medicine Diagnosis and Treatment of Stable Angina Pectoris in Coronary Heart Disease": Markedly effective: Symptoms such as chest pain and chest tightness have relieved, the angina grade has decreased by ≥2 levels, and the electrocardiogram is generally normal. Effective: Symptoms such as chest pain and chest tightness have eased, the angina grade has decreased by ≥1 level, and the electrocardiogram has shown some improvement. Ineffective: No significant changes in clinical symptoms and electrocardiogram. Total effective rate: Markedly effective + Effective is the total effective rate. |
| Chen et al.(2018) | ECG efficacy evaluation criteria: Markedly Effective: The electrocardiogram has returned to a generally normal state or has reached the condition it was in before the onset of the illness. Effective: The ST segment depression in the electrocardiogram has risen by more than 0.5 mV after treatment; the inverted T waves in the main leads have become less pronounced by 25% or more, or the T wave has changed from flattened to upright; atrioventricular or intraventricular conduction blocks have improved. Ineffective: The electrocardiogram shows no change or has worsened. |
| Gong et al.(2018) | ECG efficacy evaluation: Markedly effective: The electrocardiogram has generally returned to normal or meets normal standards. Effective: For patients with ST segment depression in the pre-treatment electrocardiogram, after treatment, the ST segment has risen by more than 0.05 mV but has not reached normal levels, the inverted T waves have become less pronounced by more than 1/4, and the flattened T waves have become upright. Ineffective: No significant improvement in electrocardiogram. Aggravated: The ST segment in the electrocardiogram has decreased by more than 0.05 mV compared to before, the inverted T waves in the main leads have become more pronounced by more than 1/4, or the flattened T waves have become inverted (or vice versa). |
| Sun et al.(2018) | The formulation is based on the relevant ECG efficacy criteria in the "Efficacy Evaluation Criteria for Angina Pectoris in Coronary Heart Disease and ECG": Markedly effective: The ST segment or inverted T waves have returned to the normal range in the resting state. Effective: The electrocardiogram shows ischemic ST segment depression with a recovery of more than 0.15 mV, the inverted T waves have changed by more than 50%, or the T waves have changed from flattened to upright. Ineffective: The electrocardiogram shows no significant change compared to pre-treatment. Total effective rate: Total effective rate = (Number of markedly effective cases + Number of effective cases) / Total number of cases × 100%. |
| Deng et al.(2018) | Efficacy criteria for ECG assessment: Markedly effective: The electrocardiogram has returned to "basically normal". Effective: The ST segment is depressed but has risen by more than 0.05 mV after treatment, or the T wave has changed from flattened to upright, or the atrioventricular conduction block has improved. Ineffective: No significant improvement in electrocardiogram. Aggravated: The ST segment has further decreased by more than 0.05 mV from its pre-treatment level, or the T wave has changed from upright to flattened, or from flattened to inverted. |
| Shi et al.(2017) | ECG efficacy evaluation criteria: Markedly effective: The ischemic changes in the electrocardiogram have largely returned to normal or meet normal standards, including the recovery of the ST segment and T waves. Effective: The ST segment in the electrocardiogram has risen by more than 0.05 mV or returned to normal; the inverted T waves have become less pronounced with a recovery of more than 25%; the flattened T waves have become upright; and the PR interval or QRS duration has improved significantly. Ineffective: No significant improvement in ischemic signs on the electrocardiogram. |
| Wang et al.(2017) | Diagnostic criteria for evaluating the efficacy of angina pectoris in coronary heart disease outlined in the "Guidelines for Clinical Research on New Traditional Chinese Medicines (2002)". And the efficacy evaluation criteria for angina pectoris in coronary heart disease and arrhythmia from the 1979 "Symposium on Integrated Traditional and Western Medicine for Treating Angina Pectoris in Coronary Heart Disease and Arrhythmia". |
| Deng et al.(2017) | The formulation is based on the "Efficacy Evaluation Criteria for Angina Pectoris in Coronary Heart Disease and ECG" proposed at the 1997 symposium on integrated traditional Chinese and Western medicine treatment of angina pectoris in coronary heart disease and arrhythmias |
| Li et al.(2015) | ECG efficacy evaluation criteria: Markedly effective: After treatment, the resting electrocardiogram has returned to normal. Effective: During treatment, the resting electrocardiogram shows the ST segment has decreased and then risen by more than 0.05 mV, the inverted T waves in the main leads have become less pronounced by more than 50%, or the T waves have changed from flattened or inverted to upright. Ineffective: The condition does not meet the criteria for marked effectiveness or effectiveness mentioned above. |
| Xie et al.(2014) | The formulation is based on the "Efficacy Evaluation Criteria for Angina Pectoris in Coronary Heart Disease and ECG" from the 1979 symposium on integrated traditional Chinese and Western medicine treatment of angina pectoris in coronary heart disease and arrhythmias |
| Jin et al.(2013) | The formulation is based on the "Clinical Research Guiding Principles for New Traditional Chinese Medicines in Treating Chest Stuffiness (Angina Pectoris in Coronary Heart Disease)" and the "Efficacy Evaluation Criteria for Angina Pectoris in Coronary Heart Disease and ECG" from the 1979 Symposium on Integrated Traditional and Western Medicine for Treating Angina Pectoris in Coronary Heart Disease and Arrhythmia |
| Zhao et al.(2013) | According to the "Efficacy Evaluation Criteria for Angina Pectoris in Coronary Heart Disease and ECG", ECG efficacy assessment is divided into three levels: Markedly effective: At the end of the treatment course, the electrocardiogram has returned to normal or basically normal state. Effective: After treatment, the ST segment has risen by more than 0.05 mV but has not reached normal levels; the inverted T waves in the main leads have become less pronounced by more than 25%, or the T waves have changed from flattened to upright; and atrioventricular or intraventricular conduction blocks have shown improvement. Ineffective: After the course of treatment, the electrocardiogram is essentially the same as before treatment, with no significant improvement. |

**Table S4 Summary of Study Characteristics Included in the Network Meta-Analysis.**

| **Author (Year)** | **Nationality** | **Participant Types** | **Trial Types** | **Sample Size(Male)** | | **Age(Years)** | | **Intervention** | | **Treatment Course** | **Follow-up Period** | **Adverse Events** | **Outcomes** |
| --- | --- | --- | --- | --- | --- | --- | --- | --- | --- | --- | --- | --- | --- |
|  |  |  |  | **Control** | **Treatment** | **Control** | **Treatment** | **Control** | **Treatment** |  |  |  |  |
| Chen et al.(2023) | China | SAP | single-center | 30(18) | 30(18)/ 30(14) | 71.30±7.50 | 68.63±9.84/ 71.57±7.89 | Antianginal therapies | EA/ Needle-embedding | 4 weeks | 8 weeks | 1 (bleeding), 1 (allergy) | a, b |
| Sun et al.(2023) | China | SAP | single-center | 40(24) | 39(19) | 59.27±5.14 | 61.33±6.07 | Acupuncture +Moxibustion | Acupuncture +Moxibustion+TCM | 8 weeks | 8 weeks | None | a |
| Zhang et al.(2023) | China | SAP | single-center | 41(23) | 40(21) | 58±7 | 59±7 | Adj therapy | Acupuncture+ Adj therapy+Moxibustion | 4 weeks | 4 weeks | NR | a, c, d |
| Li et al.(2022) | China | SAP | single-center | 25(17) | 25(15) | 47.23±4.28 | 48.04±4.21 | Acupuncture | Acupuncture+TCM | 8 weeks | 8 weeks | NR | a, c, d |
| Zheng et al.(2022) | China | SAP | single-center | 44(27) | 44(29) | 62.01±7.71 | 61.93±7.93 | TCM | Acupuncture+TCM | 2 weeks | 2 weeks | NR | a, c, d |
| Fu et al.(2022) | China | SAP | single-center | 32(16) | 33(18) | 58.51±4.95 | 58.43±4.87 | Rehab | Acupuncture+Rehab | 4 weeks | 4 weeks | NR | a, c, d |
| Jiang et al.(2021) | China | T2DM complicated with CAD and AP | single-center | 37(19) | 37(17) | 65.87±5.22 | 65.15±5.13 | Antianginal therapies | Acupuncture | 3 months | 3 months | NR | a, b, c, d, e, f |
| Du et al.(2021) | China | CSAP | single-center | 60(33) | 60(37) | 63.3±6.6 | 63.5±7.5 | Antianginal therapies | Acupuncture+TCM | 4 weeks | 4 weeks | NR | a, c, d |
| Sun et al.(2021) | China | SAP | single-center | 58(33) | 58(34) | 60.62±3.47 | 60.89±2.45 | Antianginal therapies | Acupuncture | 12 weeks | 12 weeks | NR | a, f |
| Liu et al.(2021) | China | CHD with AP | single-center | 52(29) | 52(32) | 65±4 | 65±5 | TCM | Acupuncture+TCM | 2 weeks | 2 weeks | NR | a, c, d, f |
| Zhang et al.(2021) | China | UAP | single-center | 40(30) | 40(28) | 61±6 | 62±5 | Antianginal therapies | Warm Acupuncture | 1 month | 1 month | NR | a, c, d |
| Liu et al.(2021) | China | SAP | single-center | 30(20) | 30(18) | 53±5 | 54±6 | Antianginal therapies | Acupuncture+TCM | 1 month | 1 month | None | a, c, d, e, f |
| Ding et al.(2021) | China | SAP | single-center | 40(24) | 41(24) | 62.80±6.03 | 62.59±5.73 | Antianginal therapies | Acupuncture+TCM | 1 month | 1 month | None | a, c, f |
| Wang et al.(2021) | China | T2DM with SAP | single-center | 63(40) | 64(42) | 49±10 | 50±10 | Antianginal therapies | Acupuncture | 8 weeks | 8 weeks | NR | a |
| Gao et al.(2020) | China | CHD with AP | single-center | 51(31) | 51(30) | 62.03±6.38 | 62.10±6.40 | Antianginal therapies | Acupuncture+Acupressure | 30 days | 30 days | NR | a |
| Wu et al.(2020) | China | UAP | single-center | 43(27) | 43(29) | 59.57±8.36 | 59.64±8.42 | Antianginal therapies | Acupuncture+TCM | 1 month | 1 month | NR | a |
| Ye et al.(2020) | China | SAP | single-center | 58(37) | 58(35) | 67±7 | 67±6 | Antianginal therapies | Warm Acupuncture | 1 month | 1 month | NR | a, c, d |
| Zhang et al.(2020) | China | SAP | single-center | 72(38) | 74(41) | 63.16±8.63 | 61.71±8.49 | TCM Injection | Acupuncture+TCM Injection | 2 weeks | 2 weeks | NR | a |
| Wang et al.(2019) | China | UAP | single-center | 62(39) | 62(32) | 51.28±3.12 | 53.07±2.35 | Antianginal therapies | Acupuncture+TCM Injection | 15 days | 15 days | NR | a |
| Pan et al.(2019) | China | UAP | single-center | 49(33) | 49(31) | 63±9 | 63±10 | Adj therapy | Acupuncture | 30 days | 30 days | NR | a, c, d, e |
| Wang et al.(2019) | China | SAP | single-center | 42(27) | 42(25) | 61.84±8.50 | 62.40±8.11 | Antianginal therapies | Acupuncture+TCM | 8 weeks | 8 weeks | NR | a, f |
| Zhang et al.(2019) | China | SAP | multi-center | 30 | 30/30/30 | NR | NR | Antianginal therapies | Acupuncture | 4 weeks | 4 weeks | NR | c, e |
| Wu et al.(2019) | China | CHD with AP | single-center | 46(28) | 46(27) | 66.59±6.25 | 66.55±6.23 | Antianginal therapies | Acupuncture +TCM+Moxibustion | 1 month | 1 month | 4 (vomiting), 1 (fatigue) | a, f |
| Chen et al.(2019) | China | CHD with AP | single-center | 30(13) | 30(14) | 62±5 | 62±5 | TCM | TCM+EA | 4 weeks | 4 weeks | NR | c, d |
| Li et al.(2019) | China | CHD with AP | single-center | 45(19) | 51(22) | 71.69±24.03 | 73.16±23.17 | Acupuncture | Acupuncture+TCM | 4 weeks | 4 weeks | None | c, d, e |
| Gong et al.(2018) | China | SAP | single-center | 40(22) | 40(28) | 51.25±5.48 | 53.57±6.06 | Antianginal therapies | Acupuncture+TCM | 4 weeks | 4 weeks | NR | a, b, f |
| Chen et al.(2018) | China | SAP | single-center | 48(28) | 52(35) | 66.21±2.48 | 62.38±2.23 | Antianginal therapies | Acupuncture+TCM | 4 weeks | 4 weeks | 6 ( headache, dizziness, palpitations.) | a, b |
| Sun et al.(2018) | China | senior CHD with AP complicated by HCY | single-center | 44(24) | 44(26) | 62.73±5.74 | 61.42±4.86 | Antianginal therapies | Acupuncture +Moxibustion | 3 months | 3 months | NR | a, b, c, d |
| Lu et al.(2018) | China | UAP | single-center | 43(29) | 46(30) | 51.2±6.2 | 51.9±6.5 | Antianginal therapies | Acupuncture+TCM | 30 days | 30 days | NR | a, d, e |
| Deng et al.(2018) | China | SAP | single-center | 38(22) | 38(27) | 56.49±11.36 | 57.12±9.78 | Antianginal therapies | Needle-embedding | 4 weeks | 4 weeks | NR | a, b |
| Deng et al.(2017) | China | SAP | single-center | 30(15) | 30(16) | 62.4±2.3 | 63.2±2.6 | Antianginal therapies | Acupuncture +TCM+Topical Patch | 40 days | 40 days | NR | a, b, f |
| Shi et al.(2017) | China | SAP | single-center | 35(21) | 35(20) | 60.17±5.61 | 59.97±7.06 | Antianginal therapies | Acupuncture+TCM | 4 weeks | 4 weeks | NR | a, b, f |
| Wang et al.(2017) | China | CHD with AP | multi-center | 45(30) | 45(28) | 62.11±5.42 | 61.51±4.95 | Antianginal therapies | EA | 10 days | 38 days | NR | a, b |
| Wu et al.(2017) | China | SAP | single-center | 57 | 57 | NR | NR | Antianginal therapies | Acupuncture+TCM | 2 months | 2 months | None | a, c, d |
| Yan et al.(2017) | China | UAP | single-center | 47(26) | 47(27) | 63.7±4.0 | 63.4±4.3 | Adj therapy+TCM | Acupuncture | 1 month | 1 month | 1 (nausea), 1 (fatigue) | a, c, e |
| Wang et al.(2016) | China | CSX | single-center | 40 | 40 | 53±5 | 53±5 | Antianginal therapies | Warm Acupuncture | 8 weeks | 8 weeks | 6 (stomach discomfort), 3 (liver dysfunction), 2 (bleeding) | a |
| Jia et al.(2016) | China | SAP | single-center | 30 | 30/30 | NR | NR | Antianginal therapies | Acupuncture/ Acupuncture+TCM | 4 weeks | 4 weeks | None | a |
| Fu et al.(2016) | China | AP | single-center | 35(22) | 35(21) | 56.7±10.2 | 56.4±10.5 | Antianginal therapies | Acupuncture+TCM | 2 weeks | 2 weeks | NR | a |
| Li et al.(2015) | China | UAP | multi-center | 21(5) | 30(9) | 44.7 | 46.7 | Antianginal therapies | Acupuncture | 1 month | 1 month | NR | a, b |
| Wang et al.(2015) | China | SAP | multi-center | 15(5) | 15(7)/ 15(6) | 56 | 59/57 | Healthy controls | Antianginal therapies/Acupuncture | 4 weeks | 4 weeks | NR | c, e |
| Jin et al.(2015) | China | SAP | single-center | 36(14) | 36(17) | 59.37±9.26 | 60.23±10.56 | TCM Injection+TCM | Acupuncture+TCM Injection+TCM | 21 days | 21 days | NR | a, b |
| Huang et al.(2014) | China | SAP | single-center | 20(11) | 20(7)/ 20(7) | 59.95±7.49 | 59.30±6.82/ 60.55±7.81 | Acupuncture | TCM/ Acupuncture+TCM | 4 weeks | 4 weeks | NR | a |
| Xie et al.(2014) | China | SAP | single-center | 20(11) | 20(7)/ 20(7) | 59.95±7.49 | 59.30±6.82/ 60.55±7.81 | Acupuncture | TCM/ Acupuncture+TCM | 4 weeks | 4 weeks | NR | b |
| Zhao et al.(2013) | China | UAP | single-center | 30(14) | 30(15) | 58.6±5.4 | 61.4±5.8 | Antianginal therapies | Acupuncture+TCM | 20 days | 20 days | None | a, b |
| Jin et al.(2013) | China | UAP | single-center | 36(14) | 36(16)/ 36(15) | 57.13±12.14 | 54.15±13.15/ 58.41±13.2 | Antianginal therapies | Topical Patch/ Acupuncture | NR | NR | NR | a |
| Qiu et al.(2013) | China | SAP | single-center | 40(25) | 40(24) | 62.5 | 63.2 | Antianginal therapies | Acupuncture | 19 days | 19 days | NR | a |

All patients received baseline antianginal therapies. Only additional interventions beyond the baseline treatment are listed in the table. NR, not report; TCM, Traditional Chinese Medicine;AP, Angina Pectoris; CSX, Cardiac Syndrome X; SAP, Stable Angina Pectoris; UAP, Unstable Angina Pectoris; T2DM, Type 2 Diabetes Mellitus; CSAP, Chronic Stable Angina Pectoris; CHD, Coronary Heart Disease; HCY, Homocysteine; Adj therapy, Adjunctive Therapy; EA, Electroacupuncture; a, Clinical Efficacy; b, ECG Efficacy; c, frequency of episodes; d, duration of episodes; e, nitroglycerin dosage; f, TCM syndrome score.

**Table S5 SUCRA Data for Number of Angina Attacks.**

| Treatment | SUCRA Score |
| --- | --- |
| Acupuncture | 0.7821805 |
| Acupuncture + TCM | 0.670135 |
| EA + TCM | 0.6652805 |
| Warm Acupuncture + Adj therapy | 0.554595 |
| TCM | 0.531378 |
| Rehab | 0.457818 |
| Adj therapy + TCM | 0.443289 |
| Warm Acupuncture | 0.4071495 |
| Adj therapy | 0.3983085 |
| Acupuncture + Moxibustion | 0.365162 |
| Antianginal therapies | 0.224704 |

TCM, Traditional Chinese Medicine; Adj therapy, Adjunctive Therapy; EA, Electroacupuncture.

**Table S6 SUCRA Data for Duration of Angina Attacks.**

| Treatment | SUCRA Score |
| --- | --- |
| EA + TCM | 0.9506072 |
| Acupuncture + TCM | 0.8573494 |
| TCM | 0.706335 |
| Acupuncture | 0.6081228 |
| Warm Acupuncture | 0.5087556 |
| Acupuncture + Moxibustion | 0.4884128 |
| Antianginal therapies | 0.3073656 |
| Rehab | 0.2915406 |
| Warm Acupuncture + Adj therapy | 0.1706828 |
| Adj therapy | 0.1108283 |

TCM, Traditional Chinese Medicine; Adj therapy, Adjunctive Therapy; EA, Electroacupuncture.

**Table S7 SUCRA Data for Clinical Efficacy.**

| Treatment | SUCRA Score |
| --- | --- |
| Needle Retention | 0.8214 |
| TCM + Acupuncture + Moxibustion | 0.7683 |
| TCM + Acupuncture + Topical Patch | 0.7387 |
| EA | 0.7106 |
| Acupuncture + Acupressure | 0.6921 |
| Warm Acupuncture | 0.6801 |
| Acupuncture + TCM | 0.6778 |
| Adj therapy + Warm Acupuncture | 0.5977 |
| Acupuncture | 0.5896 |
| Acupuncture + Moxibustion | 0.5395 |
| Acupuncture + TCM Injection | 0.525 |
| Topical Patch | 0.5099 |
| TCM | 0.2322 |
| TCM Injection | 0.2146 |
| Adj therapy + TCM | 0.2146 |
| Adj therapy | 0.1702 |
| Antianginal therapies | 0.1645 |
| Rehab | 0.1531 |

TCM, Traditional Chinese Medicine; Adj therapy, Adjunctive Therapy; EA, Electroacupuncture.

**Table S8 SUCRA Data for ECG Efficacy Rank.**

| Treatment | SCURA Score |
| --- | --- |
| EA | 0.92948714 |
| Needle Retention | 0.77250357 |
| TCM + Acupuncture + Topical Patch | 0.59748143 |
| Acupuncture + TCM | 0.56391643 |
| Acupuncture + Moxibustion | 0.38960714 |
| Acupuncture | 0.33421643 |
| Topical Patch | 0.33202214 |
| Antianginal therapies | 0.08076571 |

TCM, Traditional Chinese Medicine; EA, Electroacupuncture.

**Table S9 SUCRA Data for TCM symptom score.**

| Treatment | SUCRA Score |
| --- | --- |
| Acupuncture+TCM+Topical Patch | 0.7502 |
| Acupuncture+TCM | 0.731571 |
| TCM+Acupuncture+Moxibustion | 0.616694 |
| Acupuncture | 0.574747 |
| Antianginal therapies | 0.1984 |
| TCM | 0.128388 |

TCM, Traditional Chinese Medicine.

**Table S10 SUCRA Data for Nitroglycerin use.**

| Treatment | SUCRA Score |
| --- | --- |
| Acupuncture + TCM | 0.87916625 |
| Acupuncture | 0.6787475 |
| Adj therapy + TCM | 0.5001425 |
| Antianginal therapies | 0.389965 |
| Adj therapy | 0.05197875 |

TCM, Traditional Chinese Medicine; Adj therapy, Adjunctive Therapy

**Supplementary Figure**


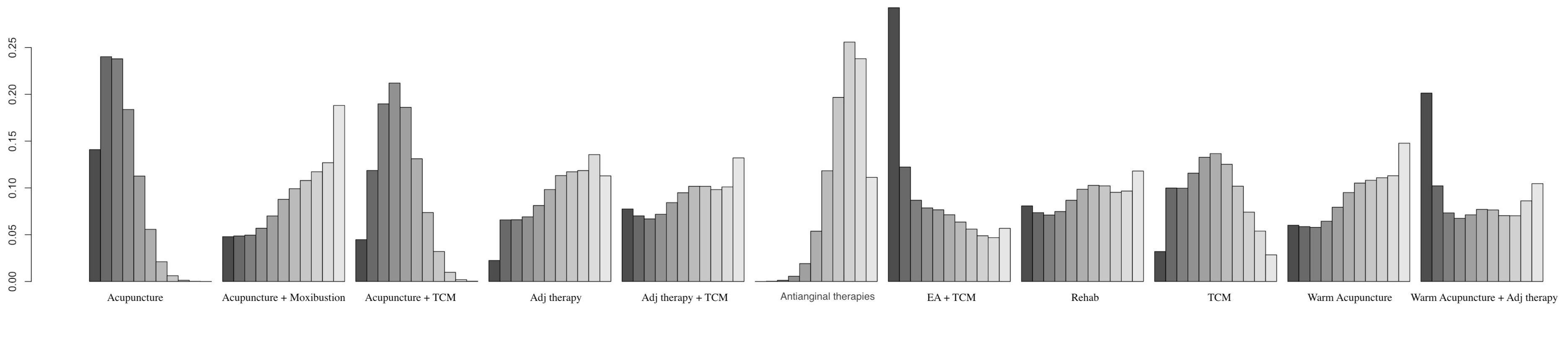


Fig S1 Rankogram for Number of Angina Attacks.


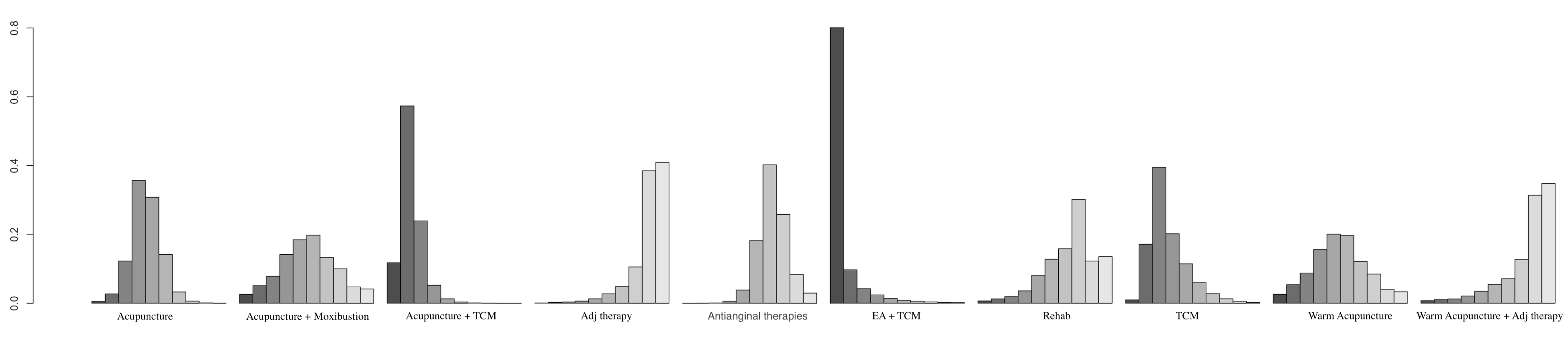


Fig S2 Rankogram for Duration of Angina Attacks.

**
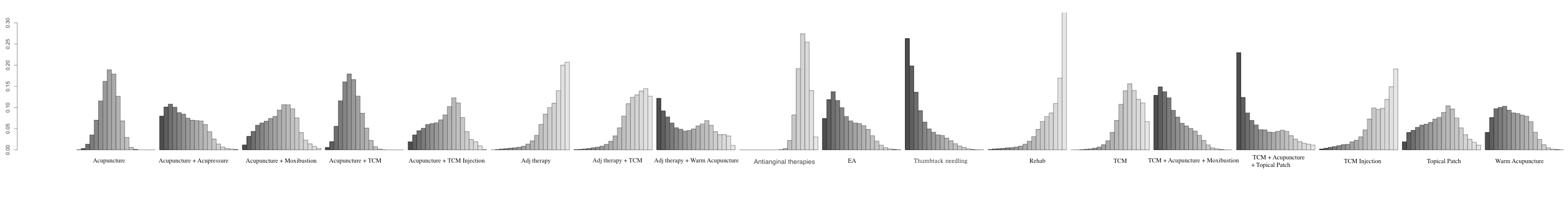
**

Fig S3 Rankogram of Clinical Efficacy.


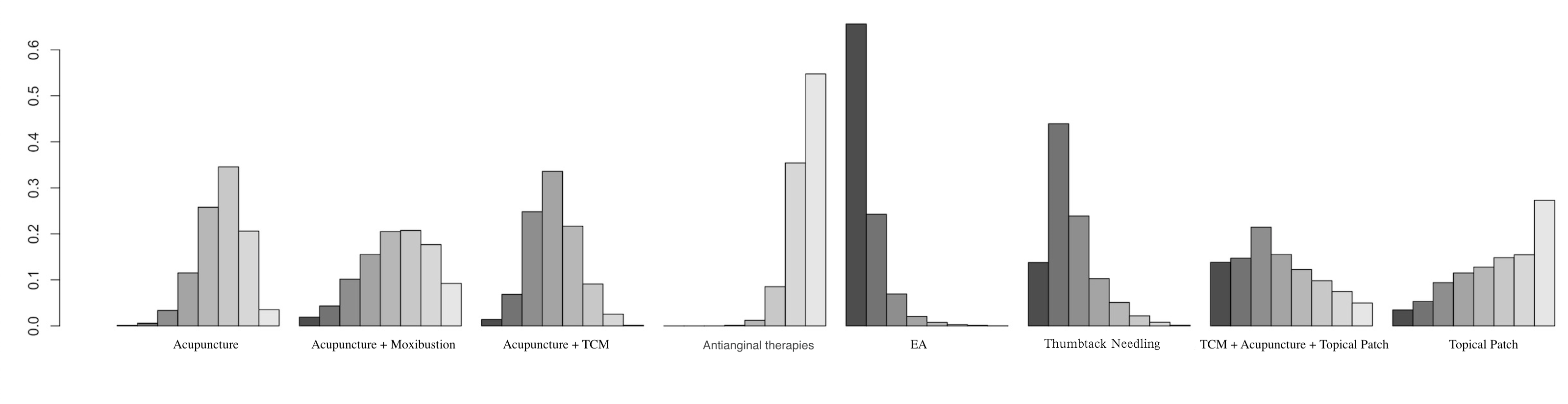


Fig S4 Rankogram of ECG Efficacy.


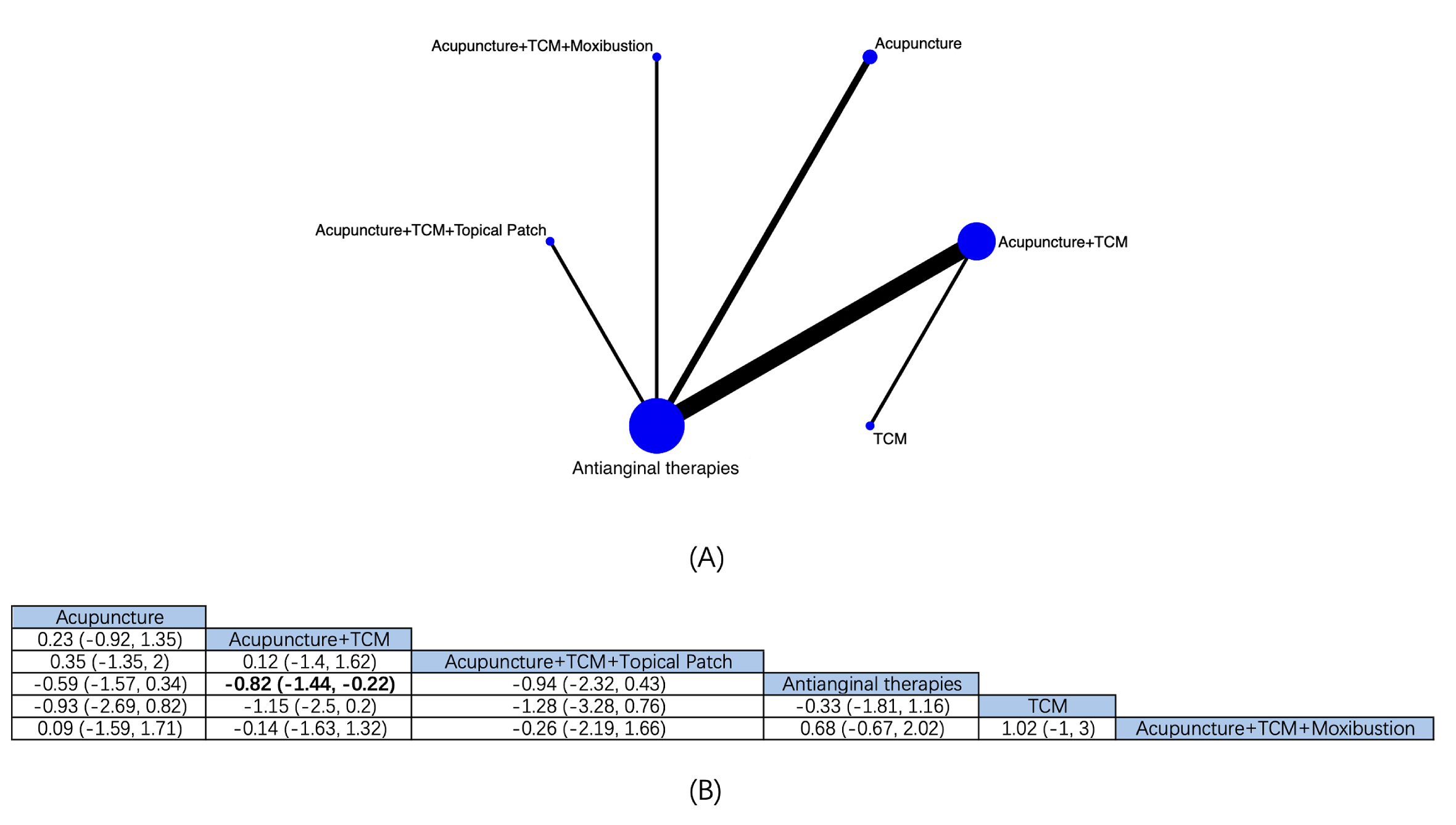


Fig S5. Network diagrams and NMA results. (A) Network plot of TCM symptom scores. (B) Relative impact of different interventions on TCM symptom scores. Note: Estimates are depicted as MD and 95% CrI. Comparisons are interpreted by reading from left to right. The estimates of treatment effects can be found at the point where the specified column intervention intersects with the specified row intervention. Noteworthy findings are highlighted in bold text.


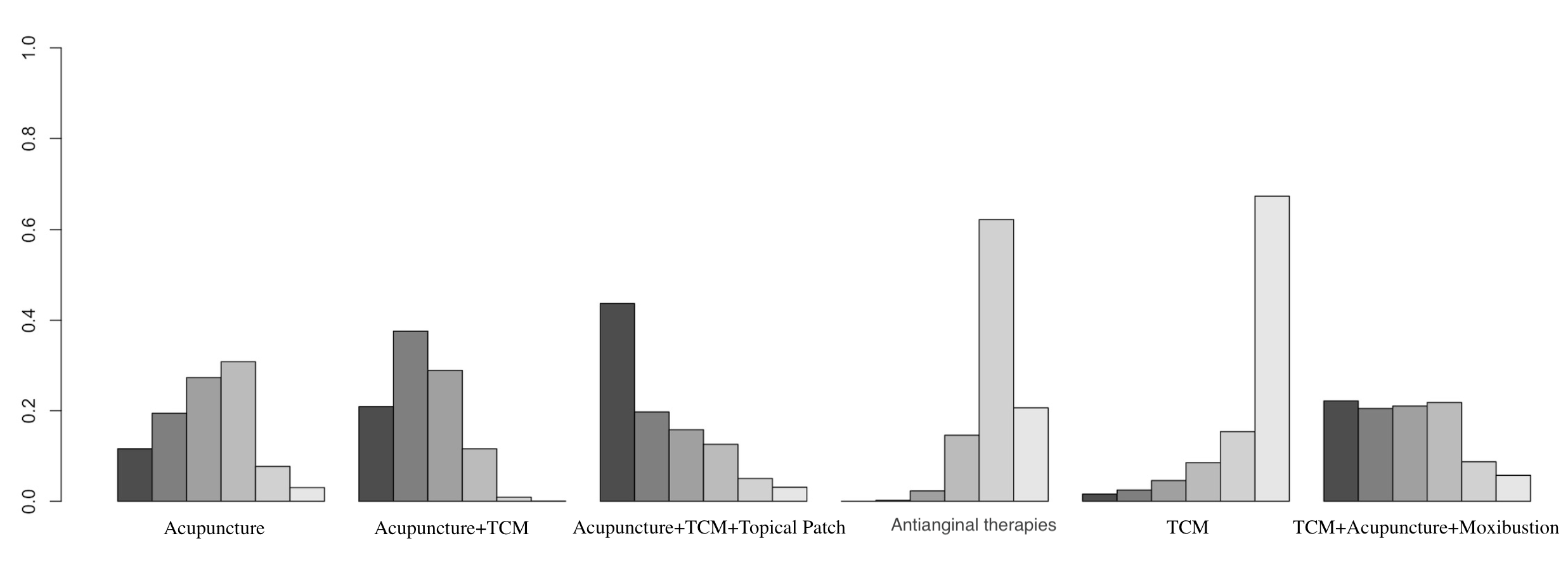


Fig S6 Rankogram for TCM symptom score.


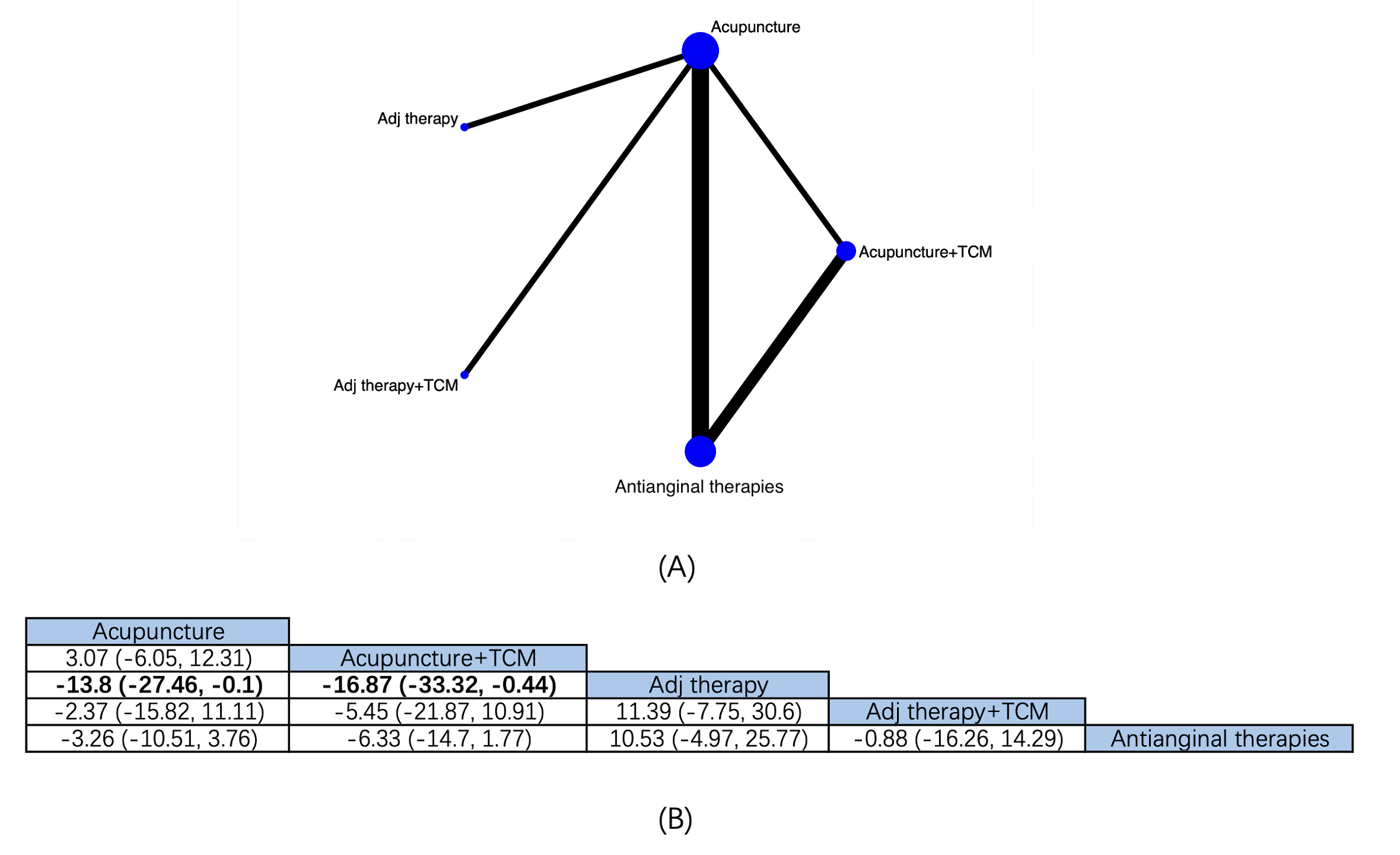


Fig S7 Network diagrams and NMA results. (A) Network plot of nitroglycerin use. (B) Relative impact of different interventions on nitroglycerin use. Note: Estimates are depicted as MD and 95% CrI. Comparisons are interpreted by reading from left to right. The estimates of treatment effects can be found at the point where the specified column intervention intersects with the specified row intervention. Noteworthy findings are highlighted in bold text.


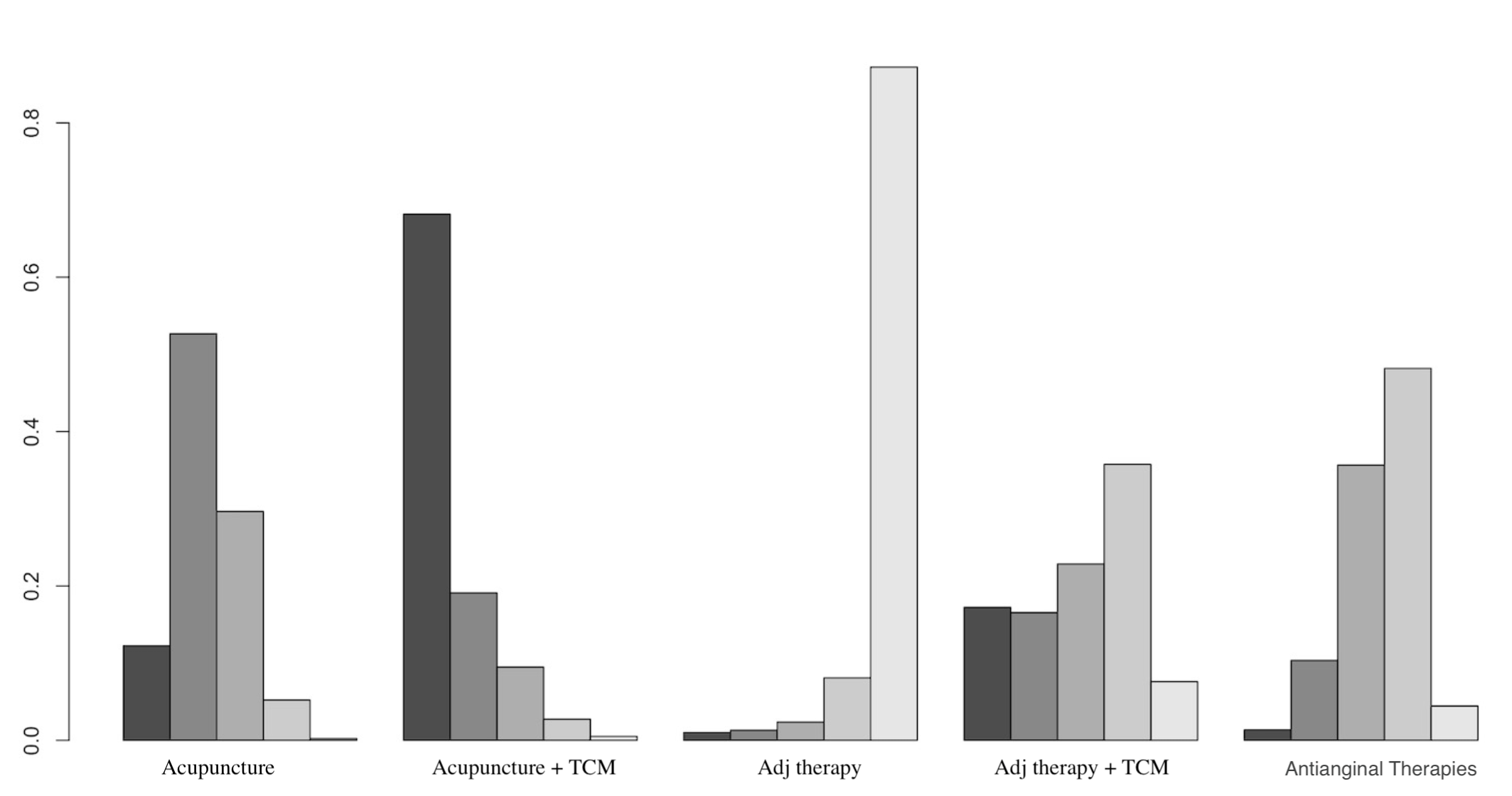


Fig S8 Rankogram for Nitroglycerin use.


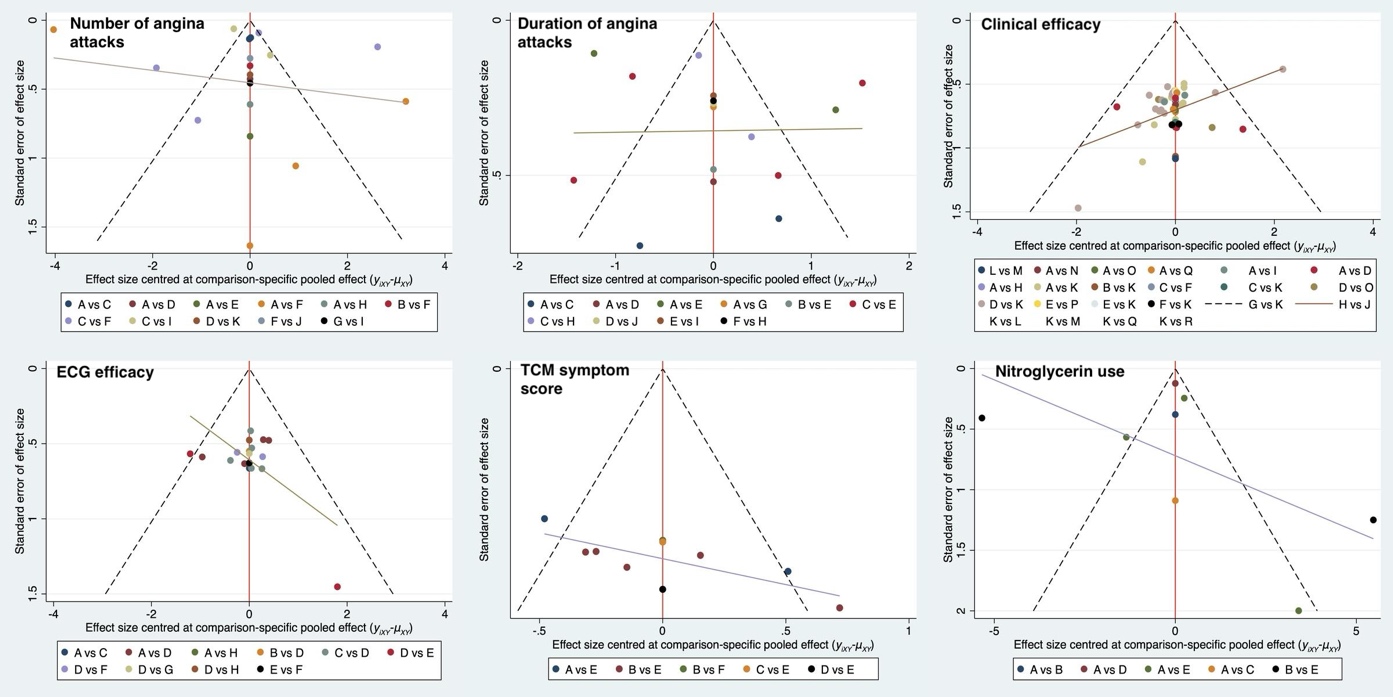


Fig S9 Publication Bias.
